# Guinea Pig (*Cavia porcellus*) Welfare: Associations Between Husbandry Practices, Human–Animal Interactions, and Animal Behaviour

**DOI:** 10.3390/ani15081157

**Published:** 2025-04-17

**Authors:** Tanja Elsbacher, Andrea Sommese, Susanne Waiblinger, Frank Künzel, Christine Arhant, Ines Windschnurer

**Affiliations:** 1 Centre for Animal Nutrition and Welfare, Clinical Department for Farm Animals and Food System Science, University of Veterinary Medicine Vienna, Veterinärplatz 1, 1210 Vienna, Austria; elsbacher_tanja@hotmail.com (T.E.); andrea.sommese@vetmeduni.ac.at (A.S.); susanne.waiblinger@vetmeduni.ac.at (S.W.); ch.arhant@gmail.com (C.A.); 2 Clinical Unit of Internal Medicine Small Animals, Special Ambulance for Small Mammals, University of Veterinary Medicine Vienna, Veterinärplatz 1, 1210 Vienna, Austria; frank.kuenzel@vetmeduni.ac.at; 3 Tierärztliche Praxis für Verhaltensmedizin Tierfairhalten, Gwörth 2, 3073 Kasten, Austria

**Keywords:** housing, feeding, care, affiliative behaviour, agonistic behaviour, repetitive behaviour, play behaviour, free roaming, enrichment, human–animal relationship, health

## Abstract

Research on the welfare of pet guinea pigs and connections between their behaviour and husbandry practices is scarce. Through an online survey among 1181 German-speaking guinea pig caretakers, we investigated husbandry practices (including social composition, housing type, free roaming, enrichment, and feeding), human–animal interactions, and guinea pig behaviour. Most participating caretakers seemed attentive to their animals’ needs and welfare, as shown by the prevalence of self-built enclosures (42.8%) and fenced floor areas (21.1%), with various enrichment and furnishings, and mostly balanced nutrition. Husbandry practices that can negatively impact welfare, such as individual housing and no constant access to hay, were observed for 7.5% and 0.4% of the animals, respectively. Eating and resting next to conspecifics and locomotor play occurred several times per day in 87.6%, 48.1%, and 19.9% of animals, respectively. Biting, running up and down and bar chewing were rare (several times per day in 0.4%, 2.9%, and 0.7% of animals, respectively). We found significant associations of guinea pig behaviour with human–animal interactions and housing conditions. For instance, affiliative behaviour and locomotor play and use of enrichment were more frequently reported in the case of more frequent positive human–animal interactions and food enrichment. Our results provide additional knowledge to support consultations on behaviour and husbandry practices.

## Simple Summary

Research on the welfare of pet guinea pigs is scarce. Proper care is essential for their well-being and healthy expression of their behaviour. We conducted a survey with guinea pig caretakers to learn about husbandry practices, such as social composition, housing type, free roaming, enrichment, and feeding. We also investigated human–animal interactions to understand how guinea pig behaviour is influenced by caretakers. Most participants seemed attentive to their animals’ needs and welfare, as shown by providing spacious self-built enclosures and larger fenced floor areas, with various enrichment, and mostly balanced diet. However, some practices can negatively impact welfare, like individual housing or not providing constant access to hay. Signs of good welfare, such as eating and resting peacefully next to conspecifics and play behaviour, like “popcorning” (locomotor play), were common. Behaviours that might indicate poor welfare were rare. We found that guinea pig behaviour was significantly associated with human–animal interactions and housing conditions. For example, friendly behaviours exchanged among animals and popcorning and use of enrichment were more frequent when caretakers interacted positively with their animals and provided food enrichment more often. Our results provide valuable insights to support behavioural consultations and consultations on optimised husbandry practices of pet guinea pigs.

## 1. Introduction

Archaeological evidence suggests that guinea pigs were domesticated in the central Andean region as early as 6000–2000 BC [1]. Introduced to Europe by Spanish colonialists in the 16th century [2], these small rodents are now among the most popular companion animals in European homes. As their popularity continues to grow [3], it is important to ensure that their care needs are met, taking into account their natural behaviour and ecological requirements.

Guinea pigs are strict herbivores and require constant access to high-quality hay and chewing material, such as twigs, to maintain both their dental and gastrointestinal health [2,4,5,6]. As prey animals, they are easily frightened and can become highly stressed if they do not have sufficient opportunities to retreat and hide when disturbed [5,7]. Their wild counterparts spend a significant portion of their time foraging and moving in areas with shrubs and grass [5,8], which also shelter them. This suggests that domestic guinea pigs benefit from having hiding places, ample space to roam, and environmental enrichment, which provides exercise, encourages locomotor play, and keeps them mentally stimulated [5]. Despite the critical impact of housing and management on the health, behaviour, and welfare of guinea pigs, there is limited information on whether the needs of guinea pigs kept as companion animals are being adequately met. This topic has recently attracted some research interest (for Germany: [9]; UK: [10,11]; New Zealand: [12,13]; Norway: [14]). However, the German study was a large-scale survey covering the housing of several exotic pets and included only a few specific questions on guinea pigs. Furthermore, findings from the UK, Norway, and New Zealand may not fully reflect husbandry practices in Central Europe.

Guinea pigs can be housed in a variety of ways, with setups ranging from cages or huts (with or without an attached exercise area) to pens (fenced indoor floor areas) or even free-range environments, such as rooms, sheds, or gardens [10]. The composition of groups also varies considerably: they are kept individually, in same-sex pairs, mixed-sex pairs, harems (1 male and 2 or more females), all-male groups, all-female groups, or small or large mixed-sex groups with up to 20 and more animals [5,15,16]. However, individual housing must be avoided due to the highly social nature of guinea pigs [5,15,17], and it is legally prohibited in such countries as Austria [18] and Switzerland [19]. Keeping all-male groups can be challenging because of the increased likelihood of agonistic (conflictual) interactions [15,20]. Similarly, maintaining very large mixed-sex groups can be demanding [21]. Certain strategies, such as early castration of males and grouping them at a young age, significantly improve the chances of successfully managing all-male or large mixed-sex groups [5,20]. Additionally, rearing conditions and early social learning play a critical role in determining the long-term compatibility and well-being of guinea pigs in group settings.

Several studies have investigated the influence of group composition and social stress on the behaviour of guinea pigs in research lab settings (e.g., [22,23,24]). For instance, males reared with only one female after being removed from a large mixed-sex colony at 30 days of age showed significantly greater aggression towards conspecifics compared to males that remained in a large mixed-sex group [22]. Moreover, early social stress due to an unstable social environment led to behavioural masculinisation in female offspring [23]. Brewer et al. [24] found that laboratory guinea pigs with regular access to a roaming area showed higher levels of activity and more social interactions than those kept exclusively in cages.

Previous studies on guinea pigs kept as companion animals mainly investigated associations between husbandry (e.g., soil conditions, feeding) and diseases, such as pododermatitis and dental diseases [3,11,25]. However, to our knowledge, only Harrup and Rooney [10] looked into associations between husbandry practices and the behaviour of guinea pigs kept as companion animals. They found that guinea pigs housed with conspecifics and provided with larger enclosures exhibited more frequent “positive behaviours”. These behaviours included lying on their side, standing on hind legs, “popcorning” (locomotor play), emitting high-pitched vocalisations (typically in anticipation of food or reward), and gnawing on items within their enclosure. In contrast, guinea pigs housed with rabbits showed fewer “positive behaviours” [10].

Beyond housing and group composition, human–animal interactions also influence the behaviour of animals (e.g., [26,27]). To our knowledge, only two studies [28,29] have investigated the combined effects of human interactions and environmental variation on guinea pig behaviour, both in the context of animal-assisted therapy. In one study [28], the behaviour of guinea pigs was observed under the following three conditions: (1) during an animal-assisted therapy session with human interaction and permanent access to a “table cage”, a home-made enclosure with a house and other hiding places, mounted on a table; (2) during a therapy session without retreat possibility (i.e., placed on a person’s lap); and (3) during a control situation without human interaction while in the “table cage”. Guinea pigs in therapy sessions with retreat possibility hid more often, but not for longer, than those in the control situation when in the table cage without human contact. While comfort behaviours remained consistent, exploratory behaviour and locomotion significantly increased in the therapy setting with a retreat possibility [28]. In the second study [29], which also investigated the effects of the availability of a retreat possibility during animal-assisted therapy sessions on guinea pig behaviour and eye temperature as indicators of stress, freezing occurred more frequently in the absence of a retreat possibility, indicating increased stress responses. In addition, prolonged stroking of the animals during the therapy session was associated with greater increases in an eye temperature, which was interpreted as stroking potentially causing stress. Despite these findings, no studies have yet examined the influence of the human–animal relationship, such as the frequency and type of human–animal interactions, on the behaviour of guinea pigs kept as companion animals.

The present study aimed to explore the current husbandry practices, behaviour, and health status of guinea pigs in German-speaking households, as well as the human–animal relationship, to gain deeper insight into the welfare of pet guinea pigs. In addition, we investigated associations between husbandry conditions, human–animal relationships, and guinea pigs’ behaviours. Our findings are intended to contribute to the development and refinement of welfare assessment protocols, improve husbandry guidelines, and promote knowledge to support behaviour consultations and care practices.

## 2. Material and Methods

The project plan and questionnaire were submitted to the ethics committee of the University of Medicine, Vienna. The committee confirmed that no ethics vote was necessary for this type of study, following guidelines for good scientific practice and with Austrian national legislation. At the start of the questionnaire, participating caretakers were provided with detailed information in the informed consent section. They were informed about data protection regulations and that the study aimed to provide an overview of the husbandry of guinea pigs, their behaviour, and the human–guinea pig relationship, as well as to investigate possible connections between these aspects. It was emphasised that all responses would be analysed anonymously and that they could discontinue their involvement at any time.

### 2.1. Questionnaire Creation and Content

Parts of the questionnaire were adapted from previous studies assessing the husbandry, health, behaviour, and human–animal relationship in ferrets and chinchillas [30,31]. Additional questions specific to guinea pigs—targeting their behaviour, health, and husbandry—were newly developed based on extensive literature research, our research questions, and discussions among experts in animal husbandry, behaviour, welfare, human–animal relationship, and companion animal medicine. To ensure clarity and completeness, five guinea pig caretakers pretested the questionnaire. This helped to identify unclear or missing elements, as well as potential issues with survey programming. Based on their feedback, ambiguities were reworded, and missing aspects were incorporated. The final questionnaire consisted of 72 questions, many with sub-questions. The number of questions participating caretakers would see and answer depended on the housing type (e.g., questions for caretakers keeping pairs or groups of guinea pigs would differ from caretakers with just one animal). In addition, there were no compulsory questions; thus, participating caretakers could skip questions if they chose so. This approach was designed to enhance the quality of responses and reduce dropout rates by avoiding reactance [32]. Fluctuating sample sizes resulting from this flexibility are reported in the results section. When more than one guinea pig was living in the household at the time of the survey, participants were instructed to base their answers only on the animal whose name came first alphabetically (referred to as A-animal in the survey, cf. [31,33]). This approach facilitated data analysis and helped ensure objective responses. In the manuscript, this animal is referred to as the “focus animal”.

The questionnaire comprised the following main sections relevant to the study:

**General information on the “focus animal”**: sex, whether the animal is neutered, current age, breed, and origin.

**Figure 1:**
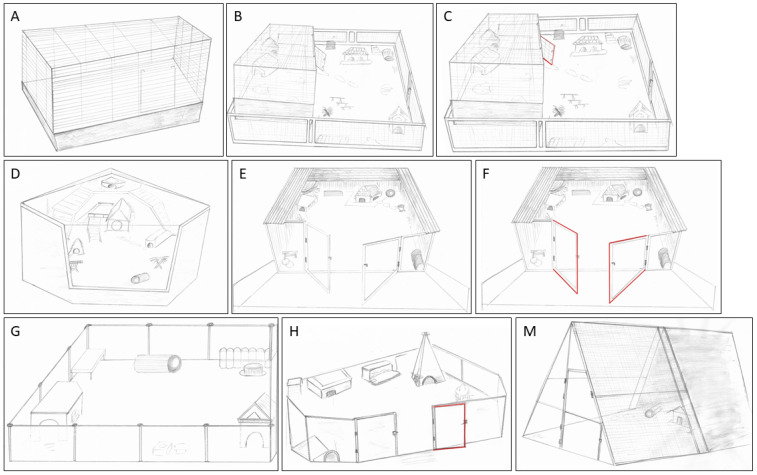
Housing types: (**A**) Cage without additional exercise possibility, (**B**) cage with temporary additional exercise possibility, (**C**) permanently open cage (door in red) with constant exercise possibility, (**D**) self-built enclosure without additional exercise possibility, (**E**) self-built enclosure with temporary additional exercise possibility, (**F**) permanently open self-built enclosure (door in red) with constant exercise possibility, (**G**) larger fenced indoor floor area without additional exercise possibility, (**H**) larger fenced indoor floor area with temporary additional exercise possibility, (I) guinea pig room (not depicted), (J) guinea pig room and temporary additional exercise possibility in other places (not depicted), (K) free flat housing (not depicted), (L) free flat housing and temporary additional roaming possibility (e.g., in cellar, or outdoors) (not depicted), (**M**) outdoor enclosure without additional exercise possibility, and (N) outdoor enclosure without additional exercise possibility (e.g., in flat, garden) (not depicted).

**Focus animal husbandry**: housing type/accommodation, with the accommodation defined as the main living area/area in which the guinea pig can stay up to 24 h a day, i.e., (almost) all the time. Exemplary housing type drawings were included in the questionnaire to help pick the right accommodation (e.g., cage, self-built enclosure, larger fenced indoor floor area, separate “guinea pig room”, in which all areas are permanently accessible to the guinea pig(s), free flat housing, outdoor enclosure, all with or without additional permanent or temporary access to an exercise area; for details see Figure 1). The drawings did not show all possibilities but were intended to assist in the correct assignment to the pre-provided options of housing types that were described in more detail. In addition, participants could describe or specify the type of housing in a comment filed in case they were not sure which housing type they should assign it to. Per definition, an exercise area could be attached directly to the accommodation or it was a separate space the guinea pig had to be brought to.

The husbandry section of the questionnaire included questions on various aspects of the guinea pig’s living environment. Participating caretakers were asked about the number of animals living together with the focus animal in the same accommodation, as well as any group size increase or reduction within the last eight weeks; in the case of solitary housing, caretakers were asked the reasons for solitary housing. Group composition, keeping with other species in the same accommodation and the frequency of presence of other species in the same room, dimensions of the accommodation, number of floor levels, availability of furnishings, frequency of providing specific enrichment, type of flooring and litter, frequency of cleaning the entire accommodation, and the changing of litter, blankets, or fleeces were also considered.

**Feeding**: frequency of feeding different food, treats, and vitamin C supplements.

**Health status and health care measures**: current health status (healthy/sick), frequency of visits to a veterinarian, and frequencies of different (health) care measures carried out by respondents (including for instance control of ears, teeth, anal region, grooming, and cleaning of the nasal environment).

**Guinea pig behaviour**: frequency of observation of specific behaviours of the focus animal within the last month (7-point score from never to several times per day), for instance, various marking behaviours, teeth chattering, hiding during roaming; frequency of observation of selected social behaviours performed by the focus animal and directed towards the focus animal within last month, for instance chasing conspecifics, resting together with conspecifics, or spraying urine at conspecifics.

**Human–animal interactions**: daily time spent engaging with the focus animal (by hand-feeding, stroking, playing, training, observing, etc.), frequency of various human–animal interactions, such as stroking, training, and playing with the focus animal.

**Demographics**: gender, age, and occupation of the caretaker, country of residence, and number of people (including children) in the household.

### 2.2. Survey and Requirements for Participation

The questionnaire was created using the online platform Survey Monkey^®^. It was accessible via a permanent link from the end of June 2020 until the end of September 2020. In the introduction, participants were instructed to ensure that only the primary caretakers of the guinea pigs participated in the survey. Eligibility required participants to currently own at least one guinea pig. The primary caretaker was defined as the individual most involved in the guinea pig’s care, including feeding and spending the most time with the focus animal. If this person was a minor, an adult was required to fill out the questionnaire alongside them. Participating caretakers could only proceed to the questionnaire after providing informed consent to participate in the study and agreeing to its terms.

### 2.3. Recruitment of Participants

The survey was advertised via social media, in German-speaking guinea pig organisations on Facebook, and on the Facebook page of the University of Veterinary Medicine. In addition, the link was sent via email to veterinarians, guinea pig organisations, the Austrian Veterinary Association, and guinea pig owners. As a means to increase motivation amongst potential participants, the option to take part in a raffle with non-cash prizes (cuddly accessories, treats, and books) was offered to participating caretakers who filled in the entire questionnaire.

### 2.4. Data Analysis

Data were imported into the statistics program IBM SPSS Statistics for Windows, Version 29.0 (IBM Corp., Armonk, NY, USA) and checked for plausibility (e.g., outliers, implausible answers). A total of 1199 responses were collected through our questionnaire. However, after plausibility checks, 18 responses had to be excluded from the analysis due to significant similarities to other submissions, suggesting potential duplications (cf. [34]). Consequently, 1181 valid questionnaires were included in the final analysis, provided that participating caretakers had answered the relevant questions. For descriptive statistics, min, max, means, standard deviations, medians, frequencies, and percentages were calculated based on completed questions. Because of the fluctuating sample size the valid sample size is always reported. Principal component analysis (PCA) was used to summarize the frequency of provision of various enrichment, (health) care measures, guinea pig behaviours (separately for social behaviours and behaviours in the main living area and during roaming), and human–animal interactions to a smaller number of components. The suitability of our data for PCA was confirmed using the Kaiser–Meyer–Olkin measure of sampling adequacy (Kaiser–Meyer–Olkin criterion ≥ 0.5) and Bartlett’s test of sphericity (*p* < 0.05). Varimax rotation was applied to help simplify the interpretation [35]. For items to be included in the respective component, their component loadings had to reach 0.4 or exceed 0.4. If an item loaded on more than one component, it needed a loading exceeding 0.6 on the component it would be included in, while the loading on the other component had to be <0.4 (cf. [31]). We reran PCAs after excluding variables with loadings below 0.4, as well as variables that loaded on more components in case they did not load higher than 0.6 on one while loading lower than 0.4 on the other(s). We decided on the number of final components for the respective PCA based on the Kaiser criterion (an Eigenvalue of at least 1.0), screen plot inspection, and component interpretability [35]. For the component labels, we considered the semantic content of the items. If only two items were included, we labelled the component after them. To allow a comparison of the components with the original scales of the items included, we calculated mean scores.

The fourteen housing types that respondents could choose from were summarized into six categories: cage, self-built enclosure, larger fenced indoor floor area, guinea pig room, free flat housing, and outdoor housing (regardless of whether constant or temporary exercise outside the main enclosure was provided) to allow us to determine whether the housing type itself makes a difference regardless of roaming possibilities. To test for differences in frequencies of reported behaviour in relation to housing types, Kruskal–Wallis tests were used, followed by Mann–Whitney U tests for post hoc testing because the dependent variables were non-parametric according to the Kolmogorov–Smirnov test.

In addition, we ran seven stepwise regression models to analyse the associations of husbandry factors (other than housing type) and human–animal interaction components as well as to focus on animal characteristics with guinea pig behaviour. Table 1 provides a detailed overview of the independent variables included in the respective model. For the dependent variables “frequency of affiliative behaviours”, “frequency of going back into enclosure and hiding during roaming”, and “frequency of locomotor play and use of enrichment” linear regression models were calculated. Through P-P plots, the normal distribution of residuals was confirmed graphically, while the homoscedasticity assumption was confirmed by plotting the standardized residuals against the standardized predicted values resulting from the model. Multicollinearity was tested for by variance inflation factor, whereas the VIF-value had to be <4.0. For “agonistic behaviours”, “competition for food”, “marking and teeth noises”, and “running up and down and bar chewing”, PCA-based subscale scores based on a frequency scale were dichotomized (occurrence no/yes) to run logistic regression models because the assumptions for linear regression models were not fulfilled. For all regression analyses for the inclusion of variables, p_entry_ = 0.10 and p_removal_ = 0.15 were set using stepwise forward selection.

For all statistical analyses, results with *p* ≤ 0.05 are referred to as significant, and with 0.05 < *p* ≤ 0.1 as a trend. When data are shown as box plots, bold lines in boxes represent the median, while the lower and upper lines of boxes represent the first and third quartiles, respectively. Whiskers represent the highest and lowest values still within the 1.5× interquartile range. Outliers (values between 1.5–3× the interquartile range) are indicated with a circle, while extreme values (outside of a range of 3× the interquartile range) are marked with an asterisk.

## 3. Results

### 3.1. Participants Characteristics

The vast majority of participating caretakers were female (96.1%), while 3.6% were male and 0.3% were gender diverse (*n* = 861). Most participants resided in Germany (71.2%), followed by Austria (26.8%), with 1.4% in Switzerland and 0.2% each in Italy and the Netherlands (*n* = 877). A small number of participants reported living in Denmark (0.1%) and Ukraine (0.1%). Regarding employment status, half of the participating caretakers were working fulltime (50.8%), while about a quarter worked half-time (23.7%), 7.6% were undergoing training/in education, and 17.9% reported to be otherwise occupied (*n* = 878). The primary participants ranged in age from 8 to 70 years old (mean ± SD: 35.6 ± 10.4 years, median: 34 years, *n* = 879). Children were present in a significant proportion of households: 24.1% had children aged 10 years or younger, while 18% had children older than 10 years or teenagers (*n* = 810). The majority of participants (85%) had heard from the survey through social networks (e.g., Facebook). Other sources included a guinea pig forum (15.9%) and acquaintances (6.2%, *n* = 881).

### 3.2. Characteristics of Focus Guinea Pigs

Approximately half of the focus animals were intact females (53%), while 2% were castrated females, 34.9% were castrated males, and 9.3% were intact males (*n* = 1118). Thus, 79.15% of males and only 3.58% of female guinea pigs were castrated. The neuter status was unknown for seven female guinea pigs (0.7%) and one male (0.1%). The most common breeds were smooth-haired (38.2%) and Abyssinian (26.1%, *n* = 1082). The guinea pigs were between 1 month and 10 years old, with an average of 3.1 ± 1.9 years (median: 3 years, *n* = 1082). They had been living under the caretakers’ care between 1 month and 10 years (mean ± SD: 2.3 ± 1.8, median: 2 years, *n* = 1087). The guinea pigs were most frequently acquired from individual people (26.9%), followed by breeders (25.9%), animal protection organisations (17.4%), pet shops (13.6%), and shelters (7.3% out of *n* = 1085). Only 3.7% of the guinea pigs had been bred by the caretakers.

### 3.3. Husbandry

#### 3.3.1. Social Environment

At the time of the survey, 7.5% of the focus animals were kept alone in their enclosure, 21.7% were kept in pairs, 18.5% were kept in a group of three, 16.1% were kept in a group of four, and the remaining 27.8% were kept in a group of five or more animals (*n* = 1043). The group size of the focus animal ranged from 1 to 81 (mean ± SD: 4.5 ± 5.3, median: 3). Among the 89 participating caretakers who housed their focus animal alone, 28 provided information on the reasons for solitary housing. Of these, 17.9% (5 out of 28) reported that the animal had been acquired from a solitary housing situation, 21.4% reported unsuccessful attempts at grouping, 3.6% (1 caretaker) noted temporarily solitary housing following castration, 25% (7 caretakers) reported that the partner animal had died, and 7.1% (2 caretakers) indicated that their guinea pigs were too old for group housing. Details on the social composition could be obtained from 918 participants. The most common group composition was a harem (46.7%), followed by mixed-sex pairs (11.8%), larger mixed-sex groups (10% with at least two males and together ≥ 5 animals), solitary housing (8.2%), and all male groups (7.2%). Male same-sex pairs were reported less often (5.7%), as were female same-sex pairs (5.2%), and all female groups (4.2%). Small mixed-sex groups (defined as more than one male with up to four animals in total) were the least common (1%, *n* = 9). Although 66.6% of caretakers stated that there was never a dog in the same room (not enclosure) with the focus animal, 11.7% stated that there were dogs in the same room several times a day (*n* = 983). For cats, 79.8% stated that there were never any cats in the same room as the focus animals, while in 8.6% of cases cats were reportedly there several times a day (*n* = 926). For more details on the frequency of the presence of dogs and cats in the same room as the focus animal, see Supplementary Materials Table S1. When asked about housing other species with their guinea pigs, 91.8% of participating caretakers (*n* = 998) reported not keeping any other species in the same enclosure. Among those who did, rabbits were most common (5.8%), followed by chinchillas (0.2%), other rodents (0.2%), and various other species (2.4%). The latter category included cats, dogs, chickens, quails, budgerigars, canaries, collared parakeets, and turtles.

#### 3.3.2. Housing Type and Free Roaming

Participating caretakers could select from fourteen housing types which were defined in the questionnaire and illustrated in sketches (see Section 2.1, Figure 1). The most commonly reported housing type was self-built enclosures without additional exercise options (27.7%), followed by larger fenced indoor floor areas without additional exercise opportunities (14.4%, *n* = 1042). For cage-based housing, cages with temporary additional exercise possibility were most frequently reported (5.7%), followed by permanently open cages with constant exercise possibility (4.4%). Cage housing in a cage without additional exercise possibility (2%) was the least common type of housing. Only a small number of guinea pigs were kept in a dedicated room with (1.4%) or without (2.3%) temporary additional exercise possibility in other places. Even fewer could move freely all over the flat, with (0.5%) or without (1.7%) the possibility of temporary additional roaming, such as cellars or outdoor spaces. When housing types were grouped into six categories (regardless of whether permanent or temporary exercise outside the main enclosure was provided), self-built enclosures were most common (42.8%, *n* = 1042), followed by larger fenced areas (21.1%), outdoor enclosures (18.0%), and cages (12.1%). Guinea pig rooms (3.7%) and free-range flat housing (2.2%) were the least common.

The total size of the main living floor area without exercise area, even if permanently accessible, measured between 0.28 m^2^ and 270 m^2^ (mean ± SD: 6.35 m^2^ ± 14.63 m^2^, median: 3.00 m^2^, *n* = 904). Cages measured, on average, 2.83 m^2^ ± 3.14 m^2^ (median: 1.60 m^2^), self-built enclosures averaged 3.59 m^2^ ± 11.63 m^2^ (median: 2.21 m^2^), and larger fenced floor areas averaged 5.73 m^2^ ± 7.46 m^2^ (median: 3.95 m^2^). Guinea pig rooms averaged 21.42 m^2^ ± 44.59 m^2^ (median: 12.00 m^2^), while for free-range flat housing, dimensions of 17.70 m^2^ ± 15.43 m^2^ (median: 11.88 m^2^) were reported, and outdoor enclosures averaged 11.45 m^2^ ± 15.44 m^2^ (median: 7.00 m^2^). When elevated and exercise areas with constant access were included in the calculation of the main living area, it ranged from 0.28 m^2^ to 818 m^2^ across all housing types (mean ± SD: 10.97 m^2^ ± 37.18 m^2^, median: 3.90 m^2^, *n* = 904). Approximately half of the guinea pigs were housed solely on the floor area (49.1%), without any elevated platforms. About a quarter (24.9%) had access to two elevated areas or platforms, and 17.7% had one elevated area. Additionally, 8.3% had three or more elevated areas (*n* = 919). Regarding exercise areas, 35.3% of the participants stated that the area was directly attached to the enclosure (but not necessarily always accessible), 32.9% indicated that the exercise area was somewhere else and that the animal had to be taken there, and 31.8% stated that they offered no additional roaming possibility (*n* = 958). Frequency of roaming was provided by 872 participating caretakers (see also Table 2). Among this sub-sample, the most common responses were no additional roaming outside the enclosure (34.7%), constant access to additional roaming area (15.1%), daily additional roaming (12.7%), and additional roaming several times per month (8.5%).

Free roaming was assessed on a 15-point scale, with 1 = “never” and 15 = “constantly” (see Table 2). Access to this opportunity was least frequently offered in guinea pigs housed in larger fenced floor areas, with a mean score of 4.53 (SD = 4.75, median: 1; *n* = 191). Guinea pigs in self-built enclosures followed closely, with a mean score of 4.88 (SD: 4.37, median score: 3, *n* = 390), which suggests roaming observed several times per month in at least 50% of the animals (since a score of 3 equalled several times per month). Those kept in dedicated rooms had more regular access, with an average of four times per week (mean: 7.11, SD: 6.07, median score: 5 (=twice per week), *n* = 27). Guinea pigs housed in cages could roam more frequently, averaging five times per week (mean: 8.22, SD: 4.39, median score: 8 (=five times a week), *n* = 101). Outdoor-housed guinea pigs had the second highest roaming frequency, with a mean score of 8.53 (SD: 5.65, *n* = 144), corresponding to approximately more than five times per week and at least 50% of the animals could roam once per day (median score: 10). For guinea pigs in free flat housing, constant roaming was assumed, reflected by a score of 15 (SD: 0, median: 15, *n* = 16).

Regarding the floor of the main living area, litter was reported most often (86.3%), followed by fleece (28.3%), plastic (13.6%), incontinence pads (13.2%), pond liner (12%), and various fabrics other than fleece (10.8%, *n* = 915). Different floor types were often combined. Wood shavings/chips/granules (72.5%), straw (15.6%), cocos litter (8.1%), hemp (7.1%), hay (4.5%), and bark/mulch (4.5%) were used most frequently as litter (*n* = 756). Some specified that litter was only used in tubs, toilet boxes, or corners.

#### 3.3.3. Furnishings and Enrichment

In terms of furnishings that were constantly available, 65% reported offering drinking dishes, with 51.7% offering nipple drinkers, 84.8% offering feeding dishes, 46.0% offering hammocks, 96.2% offering houses, 64.9% offering caves, 48.7% offering other retreats, and 79.2% offering tunnels or tubes (*n* = 943). When comparing the provision of drinking dishes and nipple drinkers, 19.0% of the focus animals reportedly had constant access to both drinking dishes and nipple drinkers, while 46.2% had only a drinking dish, 32.8% had only nipple drinkers, and 2.23% did not have constant access to either drinking dishes or nipple drinkers (*n* = 943).

Regarding the frequency of provision of various enrichment items, tunnels made of fabric, plastic, willow, wood, etc., were offered most often (at least several times per day by 69.9% of caretakers). In contrast, cat and dog toys were the least commonly offered (never by 93.7% and 92.3% of caretakers, respectively). For details, see Table 3.

A principal component analysis (PCA), was conducted to group enrichment items into components, explaining 47.7% of the total variance. Two components emerged from the analysis. The first component comprised the four items of hay ball, wooden gnawing stick, feeding tree, and fresh twigs, and was, thus, labelled “food enrichment”. The second component comprised the two items cat and dog toys and was labelled after them. Carton boxes and tunnels were not included in the final PCA because their component loadings did not reach 0.4. For further details, see Supplementary Materials Table S2.

#### 3.3.4. Human–Animal Interactions

The reported time spent engaging with the focus animal ranged from 0 to 10 h per day (average 1.1 ± 1.04 h, median: 0.5 h, *n* = 1060). Approximately half (48.7%) of the participants spent 0.5 h per day engaging with the focus animals, followed by 22.8% who reported spending 1 h and 8.7% who spent 1.5 h daily. For more detailed information, refer to the Supplementary Materials (Table S3). Regarding human–animal interactions and activities, talking was the most frequently reported activity, followed by hand-feeding (see Table 3). A PCA was performed to summarise human–animal interactions into three components, which together accounted for a total of 59.2% of the variance. The first component comprised the four items of target, clicker, trick training, and agility, and was, thus, labelled “frequency of training”. The second and third components comprised two items each and were labelled after them, “frequency of carrying around and stroking” and “frequency of talking and hand-feeding”. See the Supplementary Materials (Table S4) for further details.

#### 3.3.5. Feeding

Table 4 provides a detailed summary of food and nutritional supplements that were provided to the animals according to participants’ reports. Here, 90.3% of the focus animals constantly had access to hay and only 0.4% were reportedly never provided with hay. Feed and supplements that were offered to the focus animals of the survey mostly “never” were lime lickstones (“never” in 93.6%), bread (89.6%), salt lick (88.7%), concentrates with grains (84.4%), veterinarian food (82.0%), and nuts (81.2%). Vitamin supplements were also mostly absent from the animals’ diets (81.2%), while 13.2% received them occasionally.

### 3.4. Health Status, Care Measures, and Cleaning of Enclosures and Equipment

When asked about the current illnesses of the focus animal, 15.2% (*n* = 141) responded that it was currently ill (*n* = 929). The majority (82.2%) indicated that they took the focus animal to a veterinarian only when it had a health problem. While 2.8% reported never taking it to a veterinarian, 1.7% reported doing so less often than once a year, 5% reported doing so once a year, and 8.2% reported doing so several times a year (*n* = 923). Health care measures (including health checks) performed by guinea pig owners most frequently were anterior teeth, ear, and anal region checks (by 54% to 59% once per week; for details, see Table 5). Summarizing (health) care measures using a PCA resulted in two components with three items each. Together they explained 74.1% of the total variance. The first component comprised anterior teeth, ear, and anal region checking and was labelled “frequency of health checks”. The second component comprising cleaning of the nasal and eye region as well as fur grooming was labelled “frequency of cleaning and fur care”. For further details, see Supplementary Materials Table S5. More than half of the participating caretakers reported cleaning the entire enclosure once a week (54.9%), while 13.8% stated that they cleaned it daily. For more details on cleaning routines, see Table 5.

### 3.5. Guinea Pig Behaviour

#### 3.5.1. Social Behaviour

Behaviours, like eating simultaneously and peacefully next to conspecifics and resting together with a conspecific(s) (e.g., contact lying, sitting), were reported frequently (by 87.6% and 48.1% of caretakers several times per day; see Table 6). Resting alone was reported several times per day for approximately half of the guinea pigs (53.0%). Behaviours rarely reported included plucking out fur (never performed by a focus animal in 96.9% of cases; never occurred to focus animal in 97.3% of cases), avoiding each other (never in over 90% of cases), and fighting (never in 90.9% of cases). For a detailed breakdown, see Table 6.

Following a PCA, behaviours displayed in a social context were summarised into three components explaining 46.35% of the total variance. The first component comprised five components (hunting conspecifics, being hunted, biting conspecifics, being bitten, and fighting with conspecifics) and was labelled “frequency of agonistic behaviours”. The second component, which was labelled “frequency of affiliative behaviours”, comprised resting with conspecifics, naso-nasal contact, anogenital sniffing, playing with conspecifics, and sleeping with conspecific(s) in the same house/tube and eating simultaneously and peacefully next to conspecifics. The third component included the four items of stealing food from conspecifics, food being stolen from the focus animal by conspecifics, as well as blocking conspecifics from food or being blocked by them and was, thus, labelled “frequency of competition for food”. Items excluded because of double loadings or loadings below 0.4 were “being avoided by conspecifics”, “avoids contact with conspecifics”, “rests alone”, “fur plucked out by conspecifics”, “plucks out fur from conspecifics”, “mounts conspecific”, “mounted by conspecifics”, and “being sprayed with urine”. For further details, see Supplementary Materials Table S6.

##### Differences in Social Behaviour in Relation to Housing Types

The reported social behaviours of guinea pigs did not show significant differences across housing types, i.e., cages, self-built enclosures, larger fenced indoor floor areas, guinea pig rooms, free flat housing, or outdoor enclosures (“frequency of agonistic behaviours”: H = 7.07, *p* = 0.216, *n* = 898; “frequency of competition for food”: H = 4.44, *p* = 0.488, *n* = 898; “frequency of affiliative behaviours”: H = 6.19, *p* = 0.288, *n* = 896).

##### Associations of Social Behaviour with Husbandry, Human–Animal Interactions, and Focus Animal Characteristics

Table 7 presents the results of the regression models for the frequency of affiliative behaviour, as well as the occurrence of agonistic behaviour and competition for food. While the models for affiliative behaviours and the occurrence of competition for food were significant, the model for agonistic behaviours only showed a trend. Participants who provided more frequent food enrichment and engaged in activities, such as carrying and stroking the focus animal, more often reported a higher “frequency of affiliative behaviours”. Moreover, higher frequencies of affiliative behaviour were reported in male guinea pigs. Conversely, an increase in group size over the past eight weeks was associated with a lower frequency of affiliative behaviours. There was a trend suggesting that affiliative behaviour was more frequent in younger focus animals, and when participants provided more permanently accessible huts/caves/other, spent more daily time engaging with the focus animal, and talked and hand-feed their focus animals more often.

“Occurrence of agonistic behaviour” tended to be more likely in the case of less frequent provision of food enrichment. “Competition for food” was more likely reported in the case of less frequent carrying and stroking. In contrast, participating caretakers observing competition for food were more likely to report more frequent talking and hand-feeding. In addition, food competition was observed more likely in female focus animals.

#### 3.5.2. Behaviours Observed in the Main Living Area and During Roaming

Playing with toys and using enrichment was reported most frequently (Table 8, at least daily by 41.4% of caretakers), followed by locomotor play (“popcorning”, which was observed at least daily by 26.8% of caretakers) and hiding during free roaming (observed at least daily by 24.7% of participants). In contrast, running up and down and bar chewing at a certain cage location or between two specific places was reportedly observed least often (“never” within the last month according to 96.7% and 91.1% of the participants, respectively). Regarding marking behaviours, urine spraying was observed less frequently than marking with perianal glands (19.8% versus 31.3%). For more details, see Table 8.

Through a PCA, variables assessing the frequency of behaviours displayed by focus animals in the main living area and during roaming in the last month were summarised into four components explaining 59.5% of the total variance. The first component comprised the items of marking with perianal glands, urine spraying, teeth grinding, and teeth chattering and was labelled “frequency of marking and teeth noises”. The second component comprised the items of hiding during free roaming and trying to return to the cage/enclosure during free roaming and was named “frequency of going back into enclosure and hiding during roaming”. The third component was labelled “frequency of running up and down and bar chewing”, after the two items it comprised, and the fourth component (comprising popcorning (“jumping attacks”) and the use of toys/enrichment) was labelled “frequency of locomotor play and use of enrichment”. For further details, see Supplementary Materials Table S7.

##### Differences in General Behaviour in the Main Living Area and During Roaming in Relation to Housing Types

**Figure 2:**
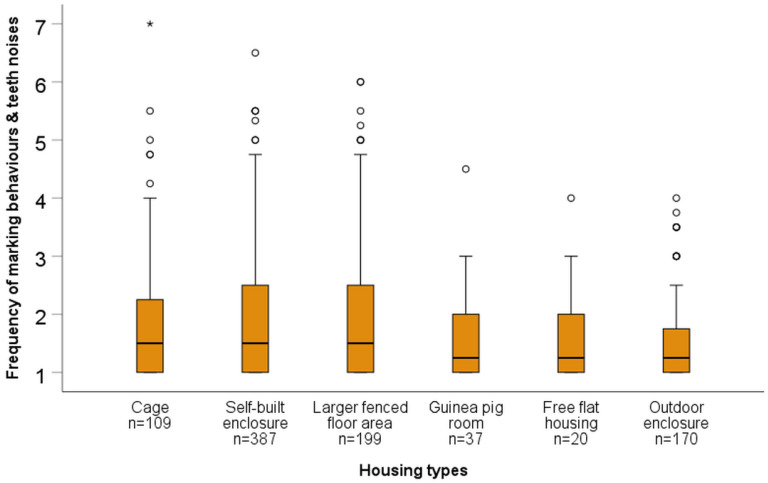
Frequency of marking behaviours and teeth noises (mean score based on principle component analyses) in relation to housing types (frequency scale: never = 1, 1×/month = 2, several times/month = 3, 1×/week = 4, several times/week = 5, 1×/day = 6, several times/day = 7). Component values were calculated by averaging the included items. Outliers are indicated with a circle, while extreme values are marked with an asterisk.

**Figure 3:**
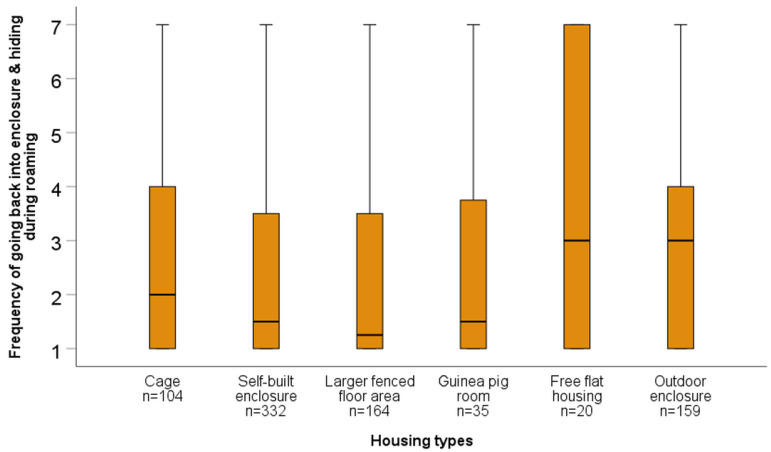
Frequency of going back into enclosure and hiding during roaming (mean score based on principle component analyses) in relation to housing types (frequency scale: never = 1, 1×/month = 2, several times/month = 3, 1×/week = 4, several times/week = 5, 1×/day = 6, several times/day = 7). Component values were calculated by averaging the included items.

**Figure 4:**
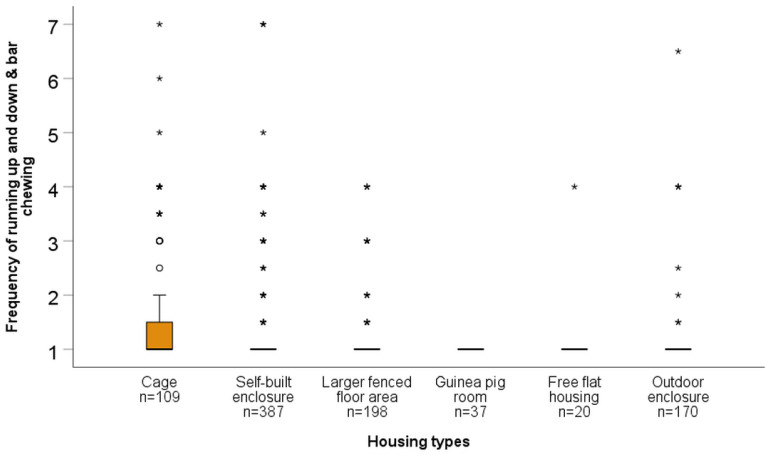
Frequency of running up and down and bar chewing (mean score based on principle component analyses) in relation to housing types (frequency scale: never = 1, 1×/month = 2, several times/month = 3, 1×/week = 4, several times/week = 5, 1×/day = 6, several times/day = 7). Component values were calculated by averaging the included items. Outliers are indicated with a circle, while extreme values are marked with an asterisk.

Kruskal–Wallis tests revealed significant overall differences in the “frequency of marking behaviours and teeth noises” (H = 28.64, *p* < 0.001, *n* = 922), “frequency of going back into enclosure and hiding during roaming” (H = 18.89, *p* = 0.002, *n* = 814), and “frequency of running up and down and bar chewing” (H = 44.66, *p* < 0.001, *n* = 921) across housing types. According to post hoc tests, “marking behaviours and teeth noises” were reported less frequently for outdoor enclosures compared to cages, self-built enclosures, and larger fenced indoor floor areas, and less often for guinea pig rooms compared to self-built enclosures or larger fenced floor areas (see Table 9, Figure 2). According to caretaker reports, “going back into enclosure and hiding during roaming” was observed more frequently in free flat housing, outdoor housing, and cage-housed guinea pigs compared to self-built enclosures and larger fenced floor areas (see Table 9, Figure 3). “Running up and down and bar chewing” were observed significantly more often in cage-housed guinea pigs compared to those housed in the other five housing types. Additionally, “running up and down and bar chewing” were significantly more frequent in guinea pigs housed in self-built enclosures or larger fenced floor areas compared to guinea pigs housed in dedicated guinea pig rooms, where such behaviours were never observed (see Table 9, Figure 4). For more details on the frequency scores for each behaviours by housing type, see Supplementary Materials Table S8.

##### Associations of General Behaviour in the Main Living Area with Husbandry, Human–Animal Interactions, and Focus Animal Characteristics

The results of the regression models for the frequency of going back into the enclosure and hiding during roaming, the frequency of locomotor play and use of enrichment, the occurrence of marking behaviour and teeth noises, and the occurrence of running up and down and bar chewing are shown in Table 10. All models were significant. A higher frequency of going back into the enclosure and hiding during roaming was reported when there were fewer animals in the same enclosure as the focus animals, when the focus animal was allowed more frequent roaming outside the enclosure, and when participating caretakers reported less frequent health checks. The frequency of locomotor play and the use of enrichment was significantly higher when there were fewer animals in the same enclosure as the focus animals, and when more frequent food enrichment, talking and hand-feeding, training, and health checks were reported. In addition, “locomotor play and use of enrichment” was reported significantly more often for younger animals. There was also a trend indicating that “locomotor play and use of enrichment” was observed more frequently when participating caretakers stated that they spent more time per day engaging with the focus animal. The occurrence of marking behaviour and teeth noises was more likely reported by participating caretakers who provided training and more frequent talking and hand-feeding. In addition, there was a trend for an increased likelihood of marking behaviour and teeth noises in cases where participants reported to spend overall more time per day engaging with the focus animal. Reports of running up and down and bar chewing were significantly more likely in the case of more frequent carrying and stroking, as well as training. Additionally, the likelihood of these behaviours tended to be higher in animals housed individually, without conspecifics in the same enclosure.

## 4. Discussion

This study aimed to explore the current husbandry, behaviour, and health status of guinea pigs in German-speaking households, as well as aspects of the human–animal relationship, to gain insight into their welfare. In addition, we investigated how housing conditions and human–animal interactions might influence guinea pig behaviour. To our knowledge, this is the first study to investigate associations between the behaviours of guinea pigs kept as companion animals and human–animal interactions. Our findings reveal that guinea pig behaviour is associated not only with housing conditions but also with human–animal interactions. Moreover, we provide a comprehensive insight into the welfare of companion guinea pigs and highlight potential welfare issues.

### 4.1. Insight into Current Husbandry, Including Human–Animal Interactions, Health Status, and the Behaviour of Guinea Pigs Kept as Companion Animals

#### 4.1.1. Social Environment

Individual housing was used for 7.5% of the focus animals, which is higher than the percentages found in German ([9]: <2%) and Norwegian samples ([14]: 3.5%). However, it is lower than the percentage of individual housing reported for New Zealand [12] and the UK [10]. Given guinea pigs’ social nature [5,15], individual housing should be avoided. In previous studies, the most common reason for individual housing were the death of a partner animal, unsuccessful attempts at grouping, and the animal’s prior solitary housing situation. These findings support the recommendation by guinea pig experts to keep at least three animals, reducing the likelihood that one animal will be left alone if a companion passes away [36]. This practice provides more time for slowly introducing new animals without one animal being completely isolated. To date, this is the first study to investigate reasons for individual housing in guinea pigs. In a similar survey among caretakers of pet rats, participants reported the same three reasons, with “lack of social compatibility” being of higher importance in rats [34].

In our study, guinea pigs were most often kept in a group of five or more animals (27.8%), closely followed by pairs (21.7%). This fits with the recommendations of experts that at least two guinea pigs should be kept together [5,16]. Our results reflect the recommended group compositions [5,16], as the harem was the most frequent structure (47%), followed by mixed-sex pairs (12%). Larger mixed-sex groups (at least two males and five or more animals altogether) were reported for only 10% of the focus animals, although such groups tend to have highly stable social structures [15]. One possible explanation for the less frequent keeping of large mixed-sex groups is the increased challenges in management monitoring for potential stress, as well as higher costs and time demands. All-male groups with three or more animals were reported by only 7% of caretakers, likely reflecting participants’ awareness of potential issues with this group composition and their tendency to avoid it [16].

In the present study, 33% of the participating caretakers reported having a dog, while 20% reported having a cat. This is similar to findings from a UK survey among guinea pig owners, where 38% of the households kept dogs and 30% kept cats. However, the UK study did not assess how often these pets had access to the guinea pigs’ main living area [10]. Cameron et al. [12] only reported that guinea pigs had access to other animals (including dogs, cats, and chickens) in just 0.9% of cases, without further details. When asked about co-housing with other species, 5.8% of participating caretakers reported keeping guinea pigs with rabbits, which is higher than the 1.7% reported in the UK survey [10]. Co-housing guinea pigs and rabbits in the same enclosure should be avoided and is forbidden in Austria [18], as it can negatively impact the guinea pigs’ welfare. Differences in social behaviour, such as rabbits’ tendency to groom each other [37,38], can create stress, as allogrooming is considerably less common in guinea pigs [5]. Rabbits can also intimidate guinea pigs, causing injuries with kicks [39]. Recent studies have shown that guinea pigs housed with rabbits display fewer “positive behaviours” indicating that such co-housing may negatively impact their welfare [10]. Additionally, rabbits can be carriers of *Bordetella bronchiseptica*, a pathogen that is often fatal to guinea pigs [4,40].

#### 4.1.2. Housing Type and Free Roaming

In our sample of German-speaking households, only 2% of focus animals were kept in a cage without additional exercise possibility, which is a considerably lower percentage than that reported in the UK [10]. This difference may be due to the higher likelihood of highly engaged and well-informed owners completing the questionnaire. Supporting this, we found that the most common housing type was a self-built enclosure without additional exercise possibility (27.7%), followed by larger fenced indoor floor area without additional exercise possibility (14.4%). Constructing an adequate accommodation requires knowledge of the animals’ needs and it is likely more time-intensive. The average floor area of the accommodations was 6.4 m^2^, significantly exceeding legal minimum requirements. For example, Austria mandates at least 0.6 m^2^ for two guinea pigs [18], Switzerland requires 0.5 m^2^ [19], and while Germany animal protection law does not specify dimensions, expert reports used in legal disputes suggest a minimum size of 2 m^2^ [41]. The Euroguide by The Federation of European Laboratory Animal Science Associations (FELASA) recommends a minimum enclosure size of 1.8 m^2^ for lab guinea pigs weighing 200–450 g, and 0.35–0.5 m^2^ per animal. For animals from 451–700 g, the enclosure should be 2.5 m^2^ with a floor area of 0.7–0.9 m^2^ per animal [42]. Given that guinea pigs are very active and have a strong drive for locomotion [5], legal minima are likely insufficient to meet their needs.

Free flat housing was the least common type of accommodation (2.2%) in our study. It is unclear whether all participants correctly understood its definition, as some reported rather small spaces averaging 18 m^2^.

Guinea pigs in our study received varying amounts of additional exercise, ranging from never (35%) to once per day (12.7%) and constantly (15.1%). Compared to the UK, where 23.8% of guinea pigs had an additional exercise area attached to their main living space [10], constant additional exercise was less frequent in our sample. This may be attributed to the differences in housing design, as the most common type in our sample was a self-built enclosure with an average floor area of 3.6 m^2^ (median 2.2 m^2^), which is relatively spacious. In comparison, Harrup and Ronney [10] reported a median surface area of 0.77 m^2^, including multi-level enclosures. The presence of multiple levels may provide greater freedom of movement, potentially promoting the perception that additional exercise is unnecessary. In our sample, 24.9% of guinea pigs had access to two levels, 17.7% had access to a single-level enclosure, and 8.3% had access to three or more levels. This contrasts with findings from Norway, where 80.3% of guinea pig enclosures had only one level [14], and New Zealand, where single-level housing was most common (54.4%), followed by two (44.4%) and three levels (1.2%) [12]. Multi-level housing can provide more usable space for guinea pigs, provided proper construction and that the animals can reach and use the levels safely (e.g., fall-proof ramps, correct angle of inclination of ramps and secured floors).

#### 4.1.3. Furnishings and Enrichment

Only 19% of caretakers provided both drinking dishes and nipple drinkers simultaneously. When acquiring new guinea pigs, it is advisable to offer multiple drinking options, such as water bowls and nipple drinkers. Guinea pigs first have to learn to drink from nipple drinkers, which in the worst case scenario can even lead to the animal’s death [39]. Moreover, dehydration can promote urolithiasis [2,4]. Hiding places were widely provided, with 96.2% of participants offering huts or other retreats. In addition to huts, various types of shelters, such as tunnels or caves, were commonly available aligning with findings from Olsen [14], where 98.9% of guinea pigs had retreat options. Cameron et al. [12] stated that 90.9% of the caretakers provided tunnel(s) and/or hideaway(s), while Bläske et al. [9] reported that retreat possibilities were provided by at least 95.9% of caretakers. As prey animals, guinea pigs instinctively seek shelter when threatened [5,36], making sufficient hiding places essential for their well-being [5]. A lack of retreat options can negatively impact their behaviour [28,29] and likely their welfare.

Guinea pigs are also neophobic and selective, often disliking sudden changes in water sources or diet [39]. However, early exposure to diverse enrichment items can help them adapt more easily to these changes [39]. This study did not look into protocols for changing furnishings, enrichment, and food.

The focus animals in this study were provided with a wide range of enrichment items, including cardboard boxes, fresh branches, tunnels, food balls, and hay balls. However, the frequency of enrichment varied considerably, ranging from occasional to constant. Enrichment, particularly in the form of feeding (e.g., hay and twigs), is not only important for stimulating natural behaviours but also plays a crucial role in maintaining health, particularly dental health. Providing structured materials, like hay, encourages prolonged chewing, which is essential for controlling the continuous growth of guinea pig teeth. The duration and intensity of chewing are more critical for proper dental wear than the hardness of the food itself [4].

#### 4.1.4. Human–Animal Interactions

The time allocated to guinea pigs is similar to or slightly lower than the durations reported for human–animal interactions with companion chinchillas and rats [31,34,43], but considerably lower than the time spent with pet rabbits [43]. Time spent with animals seems to be an important human–animal relationship measure which also relates to husbandry conditions. For rabbits, it was shown that caretakers who spent more time with their animals also kept them under better housing conditions [43].

The most common interaction reported was talking to the focus animal, which 88.2% of the participating caretakers did multiple times per day. This aligns with findings from studies on pet rats or chinchillas, where talking to the animal was also the most frequent interaction (reported by 94.9% and 79.5% of caretakers, respectively) [31,34]. The second most frequent interaction was hand-feeding, with 54.9% of caretakers doing so several times per day. Stroking was the third most frequent interaction, though it was considerably less common than talking or hand-feeding. Only 22.9% of caretakers stated that they stroke their focus animal several times per day, while 22.4% stated that they never stroked the guinea pig. Since stroking has been identified as a potential stressor for guinea pigs [29], some of the caretakers participating in our study might be unaware of its possible negative effects. However, we did not inquire about the animals’ reactions to stroking or whether owners had deliberately habituated or desensitized them to this form of interaction.

When it comes to carrying guinea pigs, 49.8% of the caretakers reported never carrying their focus animal around. In a survey on chinchillas, 63.4% of the caretakers stated that they never carried their chinchillas [31]. Gilhofer et al. [31] distinguished between caretakers who avoided carrying their animals because they assumed the chinchillas did not like being carried around (48.9%) and those who simply did not find it necessary or enjoyable (14.5%). Since guinea pigs are prey animals that generally dislike being stroked [5,36], it is reasonable to assume that, like chinchillas, they do not enjoy being carried. Therefore, it is likely that the majority of caretakers in this study refrained from carrying their guinea pigs for similar reasons. Nevertheless, lifting can be necessary to perform health checks or to carry the animals to exercise areas or if they are placed elsewhere during cleaning. Guinea pigs might learn to tolerate being carried and stroked, ideally via desensitization and counter-conditioning [5], or—if they are not generally too fearful—via habituation. Personality, general fearfulness, and prior experiences likely play a major role in trainability.

Although clicker training and target training are very good tools to provide enrichment to guinea pigs [5], very few participants in this study reported engaging in such activities. The vast majority of participating caretakers stated that they had never trained their guinea pigs in any form. Clicker training, target training, agility, or trick training were never performed by 90.3%, 92.2%, 90.0%, and 79.8% of caretakers, respectively. In contrast, training is far more common in rats kept as companion animals [34]. Similarly, in chinchillas, owners reported lower training rates, mainly because they saw no need for it or assumed their animals would not enjoy it (70.8% for clicker training and 68.9% for target training), or stated that their animal did not like it (17.3% for clicker training and 19.1% for target training). It can also be assumed that guinea pig caretakers partly do not train the animals because they see no need to do so or because they assume the animals do not like it. Further investigations into the reasons behind this reluctance, as well as promoting training as enrichment for guinea pigs, would be worthwhile, especially given the cognitive skills of guinea pigs [44,45].

#### 4.1.5. Feeding

Overall, most participating caretakers appeared well-informed about the nutritional needs of guinea pigs. The vast majority (90%) had constant access to hay, a crucial component for guinea pig health and welfare [2]. This aligns with the Norwegian study, where 93.3% of guinea pigs had continuous hay access [14]. In contrast, the UK survey reported a lower percentage (73%) [10]. In our sample, most guinea pigs (84%) never received concentrates with grains and nuts (81%), or they received these foods only occasionally. Wheat, oats, and corn are grains rich in starch and, when given in excess, disrupt the intestinal flora. Due to their high fat and protein content, seeds and nuts should be strictly avoided [4]. Other foods considered unhealthy, such as bread and processed treats, were also rarely included in their diet.

For a balanced diet, apart from constant access to hay [2], green fodder should be offered daily [5,36]. Many guinea pigs in the study likely received a diet rich enough in vitamin C. Green fodder was provided daily to 65% of the guinea pigs, a percentage comparable to the UK survey, with 70% [10]. Additionally, 91% of participants offered vegetables daily, while salad and herbs were also commonly included in their diet (daily in 62% and 32% of cases, respectively). Vitamin supplements were administered more frequently in this study than in the UK sample, with at least 19% of guinea pigs receiving them occasionally, compared to just 7% in the UK study. However, unlike the UK survey, which accounted for vitamin C supplementation through fortified pellets, the present study did not assess this aspect [10].

#### 4.1.6. Health Status, Health Care, and Maintenance Measures

Approximately 15% of the participating caretakers indicated that their guinea pig was currently ill, a higher percentage than that found in a Norwegian study (only 4.5%) [14]. This difference may be attributed to the larger sample size and broader recruitment methods used in our study. There were no marked differences in feeding, age distribution, and housing conditions between our and the Norwegian sample, except for a higher percentage of individually housed guinea pigs in our study and a greater proportion of cage-housed animals in the Norwegian study. Our findings are also not directly comparable to those of Harrup and Rooney, as their study looked at the lifetime incidence of diseases rather than the current health status of the animals. Other surveys reported by guinea pig caretakers [9,11,13] did not assess the overall current health status. Future research should investigate this further across different countries, considering such factors as genetic diversity, breed differences, and potential inbreeding.

Unlike dogs, cats, and rabbits, guinea pigs do not require regular vaccinations and they are typically taken to a veterinarian only when clear signs of illness appear [39]. In this study, 82.2% of participants stated that they seek medical care only when their guinea pigs show health problems, and 2.8% reported that they had never taken the focus animal to a veterinarian. This is a higher percentage than in a UK study, where 74.4% of the caretakers stated that they only bring their guinea pigs to a veterinarian when they think they are sick [10]. The rather irregular veterinary visits compared to other species can have various explanations. For example, Fawcett [39] suggests that the low purchase price may discourage owners from seeking veterinary care, and some owners view guinea pigs as “disposable pets”. Another possible reason for the lack of veterinary care is that the focus animal is young and might appear healthy, leading the owner to assume that a check-up is unnecessary.

More than 60% of the participants reported checking their guinea pigs’ ears, anterior teeth, and anal region at least once a week or more frequently. Regular health checks are crucial, as guinea pigs, as well as other prey species, are adept at concealing signs of illness until the condition becomes critical [46]. Owners who are well informed about the care, nutrition, and normal health of their animals are more likely to recognise early symptoms and seek veterinary care promptly, compared to those who lack knowledge or do not prioritize their pet’s well-being [39]. Maintaining a clean living environment is essential for guinea pig health, as poor hygiene can lead to the proliferation of harmful bacteria and increase the risk of disease. Given that guinea pigs have a relatively weakly muscled gastrointestinal system, they must consume food almost continuously to keep digestion moving efficiently. This results in frequent defecation and urination, which they typically release everywhere in their environment, as they cannot be reliably house-trained [5]. Consequently, guinea pig enclosures become soiled quickly and require regular cleaning. In this study, 54.9% of the participants reported cleaning the whole enclosure once a week, while 31.0% cleaned it several times a week. Only 0.3% stated that they never cleaned the entire enclosure. However, this should be further investigated, as some participants may have misunderstood the question. They might have practiced “spot cleaning” [47], i.e., removed soiled bedding and cleaned specific areas only rather than replacing all the bedding at once. In the absence of pathogens, preserving olfactory continuity might even be recommended, as guinea pigs can be sensitive to sudden environmental changes, and maintaining familiar scent profiles can contribute to their sense of security [48].

#### 4.1.7. Guinea Pig Behaviour

Regarding social behaviour, predominantly affiliative interactions were reported, while agonistic behaviours were observed rarely. When agonistic interactions did occur, they were typically mild, such as chasing or blocking access to food. More severe behaviours that could result in injuries, such as biting and fighting, were very uncommon. A retrospective study examining 1000 guinea pigs for signs of illness found bite marks in only 5 animals [25], highlighting the peaceful nature of guinea pigs towards their conspecifics.

Concerning other behaviours observed in the main living area and during roaming, repetitive behaviours, such as running up and down between specific locations and bar chewing, were reported rarely. This contrasts with findings from a UK survey, where 12% of the guinea pigs regularly showed bar gnawing [10]. Repetitive behaviour can have different causes. Stereotypical gnawing on the bars usually occurs in the same place in poor housing conditions, such as individual housing, too little enrichment, or in accommodation that is too small [5]. Also, inadequate gnawing options can lead to bars being nibbled on, and, thus, high-quality hay in unlimited quantities and suitable gnawing options should be provided [5]. However, this kind of gnawing can also occur as attention-seeking behaviour in response to anticipation of certain foods or treats [5].

Expert literature identifies the most common behavioural issues in guinea pigs as biting on bars, consuming inappropriate objects, conflicts or aggression towards conspecifics and/or people, and fur plucking [5]. These behaviours have the potential to negatively impact the human–animal relationship [49]. In the present study, undesirable behaviours, such as gnawing on furniture and urine spraying, were not reported for most animals. However, 33% of the guinea pigs had gnawed at furniture, and 20% had sprayed urine at least once in the month before the survey, with frequencies ranging from once per month to several times per day. Some participants might be highly tolerant of such behaviours, as has been observed in rabbits kept as companion animals [50], while others might prevent guinea pigs from roaming freely to avoid potential damage.

Fur plucking is described in the literature as a very rare occurrence in guinea pigs [5], which aligns with our results, as 96.9% of the focus animals never engaged in fur plucking, and 97.3% had never been subjected to fur plucking by other guinea pigs. According to Schneider [5], fur plucking is primarily associated with an ectoparasitic infestation or social instability in the group.

“Popcorning” (playful jumping) and engagement with toys and enrichment were strongly associated, loading onto a single PCA component. Therefore, they were grouped under the label “frequency of playing and use of enrichment”. Both behaviours are commonly interpreted as indicators of good welfare [51]. Popcorning was observed at least several times per week in 52% of the guinea pigs, while 59% engaged with toys and enrichment at least several times per week. Thus, suggesting that many animals showed signs of positive welfare. This is also consistent with the results from a Norwegian [14] and a UK study [10], where 59.8% or 54% of the guinea pigs exhibited similar behaviours, respectively.

### 4.2. Associations Between Husbandry, Human–Animal Relationship, and Behaviours of Guinea Pigs

#### 4.2.1. Differences in Behaviour in Relation to Housing Types

The classification of the housing type, summarised as cage housing, housing in self-constructed enclosures, housing in larger fenced indoor floor areas, guinea pig room housing, free-range flat housing, and housing in outdoor enclosures, may be insufficient to fully explain the differences in guinea pig behaviour shown in our study. We found variations in specific behaviours, including the “frequency of marking behaviours and teeth noises”, the “frequency of going back into the enclosure and hiding during roaming”, and the “frequency of running up and down and bar chewing”, which differed between housing conditions. However, no significant differences in social behaviour were found between housing types. One possible explanation is the considerable variation within each housing type, including differences in roaming opportunities, enclosure size, or stocking density.

Housing conditions significantly influenced the frequency of “going back into the enclosure and hiding during roaming”, with this behaviour occurring more often in free-range flat housing, outdoor enclosures, and cage-housed guinea pigs compared to those in self-built enclosures and larger fenced floor areas. This could also be related to the fact that the participating caretakers provided fewer opportunities for free roaming in the latter two housing types. Another explanation is that free-range flat housing and outdoor housing often provide a large total area, which may include open areas without sufficient hiding spots. As prey animals, guinea pigs require numerous hiding places [5]. In these housing systems, participants might mistakenly assume that furniture or other furnishings provide adequate hiding places. However, this might not provide the necessary sense of security and may result in animals retreating into their enclosures more frequently. Providing ample space alone is not sufficient; it must also be well-structured with appropriate hiding places to ensure that guinea pigs feel secure. A large area without adequate shelters might be underutilised, with guinea pigs showing a preference for staying close to walls, a behaviour known as thigmotaxis, or spending more time near their homes [5]. In the case of the cage-housed guinea pigs, a possible explanation for the more frequent “going back into the enclosure and hiding” could be that these animals may feel overwhelmed when outside their cage. As guinea pigs generally have less interaction with humans in the cage, they might show greater fear when exposed to a larger space and closer human proximity, resulting in more frequent retreats to their familiar spaces.

Participants reported a significantly higher occurrence of “running up and down and bar chewing” in cage-housed guinea pigs compared to those in other housing types, which may be attributed to the presence of bars, a defining feature of cage systems. Another possible explanation for this is that small cages restrict movement and limit natural behaviours, leading to frustration. Environmental stressors, such as lack of space and enrichment, are known to promote repetitive behaviours [5,52]. Interestingly, neither the enclosure size nor the roaming frequency was a significant predictor for the occurrence of “running up and down and bar chewing”. However, these were observed significantly more often in guinea pigs housed in self-built enclosures or larger fenced floor areas than in those housed in dedicated guinea pig rooms, where these behaviours were never observed. This could suggest that the presence of bars, rather than space alone, may contribute to these behaviours. Alternative explanations for no observation of “running up and down and bar chewing” in guinea pig rooms could be that guinea pigs feel more comfortable and less stressed when they are located in separated areas, or, more simply, that observation of the animals is reduced because they are housed in a separate room and are not constantly seen. In addition, self-built enclosures or larger fenced floor areas were, on average, smaller than guinea pig rooms. It should be noted, though, that cages, self-built enclosures, and larger fenced floor areas often varied considerably in size, with some providing ample space for the animals and frequent opportunities to roam. Further research, including direct observation or video analysis, is needed to clarify the underlying reasons for more “running up and down and bar chewing” in the smaller systems. The possibility that these behaviours are a form of attention seeking, particularly in anticipation of feeding [5] should also be considered.

“Marking behaviours and teeth noises” were reported less frequently in outdoor enclosures than in cages, self-built enclosures, and larger fenced floor areas. A possible explanation is that guinea pigs housed in this way are not in the same habitat as their caretakers, making it less likely that these behaviours will be observed. Teeth noises, such as the sharpening of the incisors, can be either part of the threatening behaviour of guinea pigs [5] or an expression of pain [53]. Moreover, it has been suggested that tooth chattering may occur as sign of both agitation and relaxation, and information on the behavioural context as well as the body posture are necessary for the interpretation [21]. Marking behaviours also play an important role in their communication, as guinea pigs use perianal gland secretions to mark territory and urine to mark females during courtship [5]. Similarly, “marking behaviours and teeth noises” were reported less frequently in guinea pig rooms compared to in self-built enclosures or larger fenced floor areas. As with outdoor enclosures, this may be due to a reduced likelihood of observation by caretakers, since animals housed in a separate room are not constantly heard or seen. Another explanation is that guinea pigs feel more comfortable and less stressed in a dedicated room, resulting in fewer agonistic interactions, including threats. However, there were no differences in agonistic behaviour across housing types. Additionally, marking behaviour might have been easier to observe and detect in smaller enclosures than in larger ones.

#### 4.2.2. Associations of Guinea Pig Behaviour with Husbandry Conditions, Human–Animal Interactions, and Focus Animal Characteristics

##### Associations with Social Behaviours

Several husbandry factors, human–animal interactions, and focus animal characteristics were associated with the “frequency of affiliative behaviours”. Fewer affiliative behaviours were reported when the focus animal’s group had been expanded within the previous eight weeks compared to when they were housed in stable groups. This is consistent with Sachser et al. [22], who described that guinea pigs are highly social animals that thrive in stable group structures. To minimise severe agonistic interactions, new individuals should be introduced gradually in a neutral area under direct supervision [5,17].

Participants were more likely to report affiliative behaviour when food enrichment was also provided more frequently. Enrichment engages the animals both physically and mentally, improving welfare [54,55]. In turn, improved welfare might be reflected in increased affiliative and reduced agonistic interactions, potentially through chronic stress responses affecting behaviour. Such associations have been confirmed in rats, where affiliative behaviours were more likely to occur with reduced stress or absent stress, whereas stress promoted agonistic interactions [56]. This is supported by the trend we found in the present study for a higher likelihood of agonistic behaviour with less frequent food enrichment. Food enrichment is likely a very valuable resource for guinea pigs and is especially relevant to their welfare, as it corresponds to their natural behaviour of foraging, gnawing, and continuous eating [5].

Moreover, guinea pigs tended to show more affiliative behaviours when their caretakers provided a greater number of permanently available huts, caves, and other retreats. These structures provide opportunities for animals to hide when feeling unwell or to avoid conflict with conspecifics, while a lack thereof can lead to stress and impaired well-being [5,29].

Additionally, guinea pigs whose caretakers engaged more frequently in activities including carrying, stroking, talking to, and hand-feeding were more likely to display affiliative behaviours. Similar observations have been made with dairy cows, where more positive human interactions were associated with more affiliative behaviour in the cows [57]. Increased handling and interaction may help the guinea pig to become more accustomed to the caretaker, reducing stress and improving overall welfare, which may be reflected in more affiliative behaviour. Alternatively, guinea pigs that are naturally more affiliative with conspecifics might also be more sociable with humans, making them more likely to be stroked, carried around, and engaged with. It is also possible that caretakers who spend more time engaging with their animals simply observe and, thus, report affiliative behaviour more often.

Among the guinea pig characteristics, sex significantly related to affiliative behaviour, while age seemed to follow a similar trend. Males displayed affiliative behaviour more frequently, possibly due to the fact that most were castrated, which can reduce social incompatibilities [20]. However, learned aggressive behaviour may persist [20]. Early castration, performed before sexual maturity at around three to four weeks of age, prevents males from reproducing and allows them to remain in stable groups, which in turn promotes affiliative behaviour [36].

Younger guinea pigs also showed a tendency toward more affiliative behaviour. Since the PCA-derived summary measure “frequency of affiliative behaviours” included playing with conspecifics and sleeping together in the same house or tube, this finding is consistent with previous research showing that playing is more frequent in young individuals [58] and reports that younger siblings are more likely to huddle and rest together [21]. The likelihood of the “occurrence of agonistic behaviour” tended to be higher when food enrichment was offered less frequently. In the wild, guinea pigs spend most of their time foraging for food, so providing better food enrichment contributes to improved welfare by reducing monotony and discontent [59]. This, in turn, can reduce stress and, consequently, agonistic behaviour [56].

Caregivers who observed “competition for food” were more likely to report talking to and hand-feeding their guinea pigs. Since hand-feeding can encourage competition, as guinea pigs may attempt to steal food from one another, frequent hand-feeding could increase the likelihood of observing such behaviour. To reduce such competition, it would be beneficial to provide enough food for all animals at the same time. Alternatively, the increased likelihood of food competition could be due to caretakers being more involved with their guinea pigs and observing them more closely, which increases the chance of noticing such behaviours. However, this explanation is inconsistent with our finding that competition for food was less likely to be observed when participants carried and stroked their guinea pigs more often. This suggests that even more intensive engagement did not necessarily increase the likelihood of the “occurrence of food competition”. A reason for the reduced likelihood of observing competition for food in cases of more frequent stroking and carrying around could be that these animals are generally more relaxed and less stressed, which could also relate to these animals’ personalities and general fearfulness [5].

In this study, females were more likely than males to show competition for food. As with affiliative behaviour, one reason for this could be that most of the male animals in this study were castrated (79.1%), which can reduce social tensions and make them less prone to show competition for food.

##### Associations with General Behaviour in the Main Living Area and During Roaming 

With group size and roaming frequency, two of the predictors of the “frequency of going back into enclosure and hiding during roaming” were husbandry variables. When fewer animals shared the same enclosure as the focus animal, this behaviour was reported more frequently. Since guinea pigs are highly social animals and prefer to spend their time in a group and do not feel comfortable alone [5,17,60], those with fewer companions may seek the security of their enclosure more often, especially if fewer individuals are roaming at a given time. Also the frequency of roaming outside the enclosure was a predictor. However, it showed an unexpected direction. Guinea pigs that roamed outside the enclosure also returned more often and hid during roaming more frequently. We would have expected the opposite, i.e., more fearful animals returning more often when roaming opportunities were reduced. A likely explanation is that overall increased roaming opportunities inherently lead more returns to the enclosures. In the wild, foraging periods are interrupted by the rapid seeking of shelter [5], and domestic guinea pigs may similarly prefer returning to their familiar enclosure, particularly when eating. Anecdotal evidence suggests that guinea pigs like to grab food offered to them and take it back to their main enclosure. An alternative explanation could be that caretakers who allow their animals to roam more frequently observe the animals more intensively and, therefore, notice such behaviour more often.

More frequent health checks were associated with a lower frequency of returning to the enclosure and hiding. Regular health checks might habituate guinea pigs to the caretakers, making them feel more comfortable or at least less fearful while roaming in the proximity of humans. Therefore, they might return to their enclosures and hide less often. This effect would require that such health checks are not perceived as aversive, allowing the animals to build trust with their caretakers rather than developing fear.

Several husbandry and human–animal interaction variables, as well as individual characteristics, related to the “frequency of locomotor play and use of enrichment”. The number of animals in the same enclosure as the focus animal related to the frequency of locomotor play and the use of enrichment. If there were fewer animals in the same enclosure, the “frequency of locomotor play and use of enrichment” was significantly higher. While large groups can foster stable subgroups in which animals feel comfortable [22], managing them can be more challenging. Overstocking increases the risk of resource competition and social instability, both of which can induce stress. Stress, in turn, can reduce play behaviour [61].

The importance of enrichment is underlined by the fact that the frequency of food enrichment provision was a significant predictor for locomotor play and the use of enrichment. The guinea pigs showed a significantly higher frequency of locomotor play and use of enrichment with more frequent provision of food enrichment. This may be explained not only by the fact that more enrichment increases the likelihood of enrichment use, but also by the fact that play might be affected. Food enrichment is an important part of keeping animals occupied and preventing boredom [59] and promotes well-being, which may be reflected in more frequent play behaviour [61].

In addition, human–animal interaction variables were significant predictors for locomotor play and use of enrichment. The more frequently that participating caretakers reported talking to the guinea pigs, feeding them by hand, and training them, the more often the focus animal showed play behaviour and used enrichment items. There was also a trend for a higher frequency of locomotor play and use of enrichment with more daily time spent engaging with the focus animal. Frequent positive or at least neutral human–animal interactions allow animals to habituate to their caretakers or even to develop a positive animal–human relationship, leading to reduced fear and increased comfort in their presence [62]. This is reflected in less fear of humans or animals feeling (more) comfortable around them [62]. Talking to and hand-feeding guinea pigs are considered effective ways to communicate built trust with these rather shy animals [5].

Another good way to interact with guinea pigs, as well as an activity and enrichment option, is training, which is supported by our results. Clicker training, conditioning, and learning commands can be fun for both the human and the guinea pigs if performed correctly [5]. If the animals are sufficiently occupied, this could lead to better welfare, which can be reflected in increased play behaviour and exploration [61]. However, it is crucial to monitor their behavioural responses and ensure they have control and predictability in interactions [51,63], including the option to retreat when needed, which can help reduce stress [28,29].

More frequent health checks were also related to a significantly higher frequency of locomotor play and use of enrichment. In addition to allowing the guinea pigs to become accustomed to being touched and handled by humans, regular health checks help to detect health problems early and, thus, increase the likelihood that the animals will stay healthy. Healthy animals will feel more comfortable than sick animals. Usually, activity and the frequency of playing are negatively affected in case of illnesses [61]. Finally, the younger age of the focus animals related to more frequent locomotor play and use of enrichment. As discussed for affiliative behaviour, which includes social play, it is expected that locomotor play will also occur more frequently in younger animals [58].

In the model predicting the “occurrence of marking behaviour and teeth noises”, only human–animal interaction variables were significant predictors. These behaviours were more likely reported in the case of more frequent training, talking, and hand-feeding. Moreover, there was a positive trend regarding the daily time spent engaging with the focus animal. The most likely explanation for this is that caretakers who spend more time with the animals might be better able to observe behaviours, such as marking behaviour and teeth noises. However, another explanation is that the animals perceived these interactions as aversive, which was reflected in the teeth noises, which animals make during agonistic interactions [5]. Perhaps both explanations are true, and the relationship is not linear, but depends instead on the degree of fear or trust in people. On the one hand, animals with a good human–animal relationship might receive more frequent interactions and caretakers might be more likely to observe their behaviour, including marking and teeth noises in more detail. On the other hand, caretakers of very fearful animals might try to improve the human–animal relationship by offering food and talking to them, or training them. However, this might provoke agonistic behaviour, such as certain teeth noises.

Running up and down and bar chewing are listed among behavioural problems of guinea pigs. However, both cannot only be an expression of a stereotypic behaviour; they can also reflect displacement behaviour or represent attention-seeking behaviour [5]. When the participants carried and stroked the focus animals more often, the guinea pigs were more likely to run up and down and chew on the bars more often. This could either be a sign of stress, as guinea pigs are not animals that like to be stroked [5], or it could be displacement behaviour in expectance of impending feeding. They were also more likely to show these behaviours with increasing frequency of training. As training in guinea pigs is primarily conducted with food as a motivator, displacement behaviour in relation to food anticipation and frustration might be the most likely explanation. As guinea pigs are highly social animals [5,22], it is not surprising that the likelihood of potential repetitive behaviours, such as running up and down and bar chewing, tended to be higher in our study when the animals were housed individually and, thus, deprived of companionship. Hence, the interpretation of repetitive behaviour due to social deprivation is a likely explanation in this case. However, this does not exclude attention seeking as a likely explanation, as animals housed without conspecifics might try to engage even more with their caretakers. A study in group-reared heifers that were tested either individually or with their pen mates in a novel enclosure showed that some of the isolated animals approached a stationary person, while those in a group never did [64]. To shed light on underlying reasons for “running up and down and bar chewing”, on-site observations, ideally based on detailed video analysis, are necessary.

Overall, better housing conditions (such as a greater number of retreats or hiding possibilities and more frequent food enrichment) and more positive human–animal interactions were associated with more frequent reports of behavioural indicators of positive welfare, including affiliative and play behaviour [51,65]. The importance of the social environment in terms of group stability is also underlined by the present study, with affiliative behaviour occurring more frequently in stable groups. Moreover, enrichment tended to reduce the likelihood of the occurrence of agonistic behaviour. Agonistic behaviour, particularly aggression, is used as an indicator of negative welfare in several species, as more frequent or severe aggression can result in stress and injuries (e.g., [66,67,68,69,70]). Further research is needed to clarify the relationship between more frequent positive human–animal interactions and higher frequencies of affiliative behaviour in guinea pigs.

### 4.3. Limitations

Limitations inherent to studies based on surveys among self-selected samples of participating caretakers are that the results might not be representative of all participants. It is often assumed that more engaged and committed caretakers are more likely to take the time to participate in such studies [27,31,34]. This can to some extent explain why the overall welfare of the guinea pigs seemed to be very good. Nonetheless, potential welfare issues have been identified. Another point of criticism could be that the study relied on caretaker reports, which might be inaccurate to some extent for various reasons, such as misunderstood questions resulting in conflicting information [71] or biases in self-reported behaviour [72]. Self-reports may be less accurate for a number of different reasons, including memory and social desirability biases, leading to over- or underreporting of behaviours (for a review, see [72]). However, self-reports have several advantages, such as flexibility, low cost, and, in particular, the possibility of investigating behaviours that cannot be observed otherwise without a considerable amount of effort, e.g., infrequent behaviours [72]. Also, the fact that animal behaviour was reported by the participating caretakers, rather that direct or video observation by trained observers, could to some extent introduce biases due to subjective views. We tried to avoid this by describing the guinea pig behaviours inquired after in the questionnaire as objectively as possible, similar to an ethogram, without interpreting it. Despite criticism questioning their validity [73] assessments of participants provide very valuable data, since they know their animals best. Clearly, follow-up studies with observations of guinea pigs in their home environment would be interesting.

A limitation of not having a forced response is the fluctuating sample size. However, a forced response option would have increased the risk of complete dropout of participants and could have reduced the quality of the answers [32]. While we acknowledge that some of the findings we reported are based on trends, we believe that these insights are valuable for future research. Studies with larger sample sizes or direct behavioural observations could further investigate these patterns, helping to confirm or refine our conclusions. Finally, as is characteristic of epidemiological data collection, we could only test for significant associations. Thus, due to the very nature of the study, no conclusions on causal relationships can be drawn.

## 5. Conclusions

Our study provides extensive insight into guinea pig welfare in German-speaking households, including husbandry practices, human–animal interactions, and animal behaviour that might reflect good or impaired welfare. In summary, the majority of participating caretakers appear well-informed about aspects relevant to the welfare of their guinea pigs. Nevertheless, there is still room for improvement, as 7.5% of the guinea pigs were still housed individually. Interestingly, a sizable percentage of participants reported never to provide additional exercise. Further clarification would be needed as to whether these participants considered the size of their guinea pigs’ enclosure to be sufficient. An interesting finding regarding human–animal interactions was that some caretakers stroke their guinea pigs frequently, although it has been suggested that such animals do not enjoy stroking. Since the animals’ reactions during or after the stroking sessions were not examined, but more frequent carrying and stroking related to more frequent affiliative behaviour and an increased likelihood for the observation of running up and down and bar chewing, further investigations would be necessary. Because many participants only take their focus animal to the veterinarian when necessary, and more than one-sixth of the focus animals were ill at the time of the survey, owner education to encourage annual check-ups would be necessary. Affiliative and play behaviour seem to be also useful indicators of good welfare in guinea pigs because they were observed more often in cases with more food enrichment and more frequent positive human–animal interactions. For running up and down and bar chewing, which were rarely reported, the interpretation of these behaviours as repetitive behaviours and, thus, an indicator of impaired welfare, is not straightforward. In some animals, it might rather be a means of attention seeking or a form of displacement behaviour while waiting for food. Overall, our findings promote the understanding of housing conditions and the behaviour of guinea pigs kept as pets. Further studies including observations of animals are warranted to confirm our findings and to investigate the new questions that have arisen.

## Figures and Tables

**Table 1:** Overview of dependent variables (frequencies and occurrence no/yes) and independent variables of the linear and ordinal regression models. The same independent variables were always entered as predictors in the three models for social behaviour and the four models for behaviours in the main living area and during roaming.

Dependent Variables	Included Independent Variables
Frequency of affiliative behaviours Occurrence of agonistic behaviours (no/yes) Occurrence of competition for food (no/yes)	Size of the enclosure/accommodation area including elevated areas and exercise areas in case of constant access Roaming frequency outside the enclosure Number of permanently accessible huts/caves/other Food enrichment Cat/dog toys Number of animals in the same enclosure as the focus animal Group size increase within the prior 8 weeks no (0) yes (1) Group size decrease within the prior 8 weeks no (0) yes (1) Daily time spent engaging with focus animal Frequency of lifting up Frequency of carrying and stroking Frequency of talking hand-feeding Female (0) vs. male (1) Age of focus animal
Frequency of going back into enclosure and hiding during roaming Frequency of locomotor play and use of enrichment Occurrence of marking behaviour and teeth noises (no/yes) Occurrence of running up and down and bar chewing (no/yes)	Size of the enclosure/accommodation area including elevated areas and exercise areas in case of constant access Roaming frequency outside the enclosure Number of permanently accessible huts/caves/other Food enrichment Cat/dog toys Individual housing yes (0) no (1) Number of animals in the same enclosure as focus animal Group size increase within the prior 8 weeks no (0) yes (1) Group size decrease within the prior 8 weeks no (0) yes (1) Frequency of presence of dogs in the same room Frequency of presence of cats in the same room Daily time spent engaging with focus animal Frequency of lifting up Frequency of carrying and stroking Frequency of talking hand-feeding Frequency of training Frequency of health checks Frequency of cleaning and fur care Duration of residence of the focus animal at the caretaker’s Age of focus animal (only included in the frequency of locomotor play and use of enrichment)

**Table 2:** Roaming frequency outside the enclosure was assessed using a 15-point score from 1 (never) to 15 (constantly).

Frequency of Roaming Offered	*n*	%
Never	303	34.7
1×/month	23	2.6
Several times per month	74	8.5
1×/week	36	4.1
2×/week	37	4.2
3×/week	38	4.4
4×/week	37	4.2
5×/week	31	3.6
6×/week	18	2.1
1×/day	111	12.7
2×/day	19	2.2
3×/day	0	0
4×/day	1	0.1
More than 4×/day	12	1.4
Constantly	132	15.1

**Table 3:** Heat map providing an overview of the frequency of offering different enrichment items (responses on a 7-point scale from never to constantly) and human–animal interactions (responses on an 8-point scale from never to several times/say). Frequencies are depicted in the percentage of responses (*n*). The shade of orange darkens with increasing percentages.

Enrichment Provided	*n*	Never	Occasionally	1×/Week	Several Times/Week	1×/Day	Several Times/Day	Constantly	
Cardboard box	923	39.3%	42.1%	3.6%	5.3%	0.7%	0.8%	8.2%	
Logic toy	915	55.7%	28.2%	5.0%	7.1%	0.7%	0.2%	3.1%	
Nibble wood	898	54.1%	27.2%	4.2%	4.8%	1.0%	0.3%	8.4%	
Fresh branches	938	5.4%	35.1%	15.4%	25.1%	4.5%	1.2%	13.4%	
Tunnel made of fabric, plastic, willow, wood, etc.	930	7.0%	13.9%	1.8%	5.8%	1.6%	3.4%	66.5%	
Feeding tree (log with holes to be filled, attached to a wooden plate)	919	66.9%	19.9%	2.9%	3.8%	0.9%	0.4%	5.1%	
Dog toys	918	93.7%	5.0%	0.3%	0.2%	0.1%	0.0%	0.7%	
Cat toys	921	92.3%	5.5%	0.2%	0.3%	0.1%	0.2%	1.3%	
Food ball	923	56.6%	29.7%	3.5%	5.2%	0.8%	0.4%	3.9%	
Hay ball	928	52.9%	33.2%	2.4%	2.9%	1.0%	0.5%	7.1%	
**Human–Animal Interactions**	***n***	**Never**	**Occasionally**	**1×/Month**	**Several Times/Month**	**1×/Week**	**Several Times/Week**	**1×/Day**	**Several Times/Day**
Stroking	935	22.4%	31.2%	0.6%	2.1%	2.8%	8.3%	9.6%	22.9%
Talking	942	0.5%	3.4%	0.0%	0.1%	0.1%	2.3%	5.3%	88.2%
Clicker training	924	90.3%	5.8%	0.2%	0.8%	0.4%	1.1%	0.9%	0.5%
Target training	923	92.2%	4.7%	0.0%	0.5%	0.4%	1.1%	0.5%	0.5%
Agility	922	90.0%	6.2%	0.2%	0.8%	0.4%	0.5%	1.0%	0.9%
Trick training	924	79.8%	11.9%	0.0%	0.9%	1.3%	2.6%	1.4%	2.2%
Hand-feeding	940	2.0%	8.2%	0.1%	1.2%	1.3%	12.4%	19.9%	54.9%
Carrying around	932	49.8%	22.7%	1.4%	2.0%	7.2%	6.5%	6.2%	4.1%

**Table 4:** Heat map giving an overview of the frequency of provision of different foods and supplements. Frequencies are depicted in the percentage of responses (*n*). The shade of orange darkens with an increasing percentages.

Food and Supplements	*n*	Never	Occasionally	1×/Week	Several Times/ Week	1×/Day	Several Times/ Day	Constant Access
Hay	940	0.4%	1.3%	0.1%	1.1%	2.3%	4.5%	90.3%
Straw	925	39.5%	25.6%	3.4%	3.2%	0.4%	1.1%	26.8%
Dried herbs	940	3.3%	26.0%	11.7%	21.0%	10.2%	4.5%	23.4%
Vegetable flakes	936	12.3%	31.3%	8.7%	18.1%	17.6%	6.3%	5.8%
Hay pellets	929	56.8%	25.4%	3.2%	4.5%	3.6%	1.5%	5.0%
Concentrates with grains	928	84.4%	8.4%	0.9%	1.6%	1.6%	0.2%	2.9%
Compound feed without grains	926	39.3%	28.3%	5.5%	7.7%	8.0%	1.0%	10.3%
Nuts	930	81.2%	15.5%	1.1%	0.6%	0.5%	0.2%	0.9%
Veterinarian food	920	82.0%	14.5%	0.7%	0.9%	1.1%	0.4%	0.5%
Green fodder	937	0.9%	11.5%	3.4%	19.3%	16.5%	26.6%	21.8%
Vegetables	941	0.0%	2.1%	0.5%	6.0%	23.6%	46.1%	21.7%
Culinary herbs	935	2.8%	22.0%	8.3%	34.9%	12.5%	11.4%	8.0%
Fruit	937	5.3%	48.8%	17.7%	16.6%	6.4%	2.7%	2.5%
Salad	940	1.7%	11.6%	2.9%	21.5%	18.0%	28.0%	16.4%
Branches leaves	938	6.3%	35.9%	12.8%	24.0%	5.7%	1.8%	13.5%
Bread	940	89.6%	8.3%	0.6%	0.5%	0.3%	0.0%	0.6%
Treats	936	59.9%	27.9%	2.6%	2.9%	4.1%	2.4%	0.3%
Lime lickstone	935	93.6%	3.1%	0.2%	0.1%	0.0%	0.0%	3.0%
Salt lick	934	88.7%	3.5%	0.1%	0.0%	0.0%	0.0%	7.7%
Nibble sticks	934	64.7%	29.0%	2.8%	1.2%	0.3%	0.0%	2.0%
Nibble woods	935	68.1%	25.3%	1.3%	1.9%	0.1%	0.0%	3.2%
Vitamin supplements	934	81.2%	13.2%	2.0%	1.2%	1.9%	0.2%	0.3%

**Table 5:** Heat map giving an overview of health care measures and cleaning routines. Frequencies are depicted in percentage of responses (*n*). The shade of orange darkens with increasing percentages.

(Health) Care Measures	*n*	Never	Occasionally	1×/ Month	Several Times/Month	1×/ Week	Several Times/Week	Daily	
Nail clipping	928	4.6%	29.5%	47.2%	0.0%	18.3%	0.2%	0.1%	
Ear check	929	1.8%	18.1%	16.9%	0.0%	56.9%	4.0%	2.3%	
Anterior teeth check	928	2.3%	15.8%	17.1%	0.0%	58.6%	3.2%	2.9%	
Fur grooming	926	40.4%	24.6%	7.6%	0.0%	22.6%	3.6%	1.3%	
Anal region check	927	2.7%	15.6%	12.6%	0.0%	54.0%	8.4%	6.6%	
Cleaning eye region	923	37.4%	32.1%	4.2%	0.0%	18.9%	3.8%	3.7%	
Cleaning nasal region	919	46.1%	29.1%	3.3%	0.0%	16.6%	2.9%	2.0%	
**Cleaning of the Following Areas**	***n***	**Never**	**Occasionally**	**1×/** **Month**	**Several Times/Month**	**1×/** **Week**	**2×/Week**	**>2×/Week**	**Daily**
Cleaning whole enclosure	929	0.3%	0.0%	0.0%	0.0%	54.9%	31.0%	0.0%	13.8%
Cleaning the whole exercise area	750	11.1%	0.0%	0.0%	0.0%	46.8%	23.7%	0.0%	18.4%
Litter only	817	7.0%	0.0%	0.0%	0.0%	43.6%	26.7%	0.0%	22.8%
Blankets/fleece (incontinence pads, etc.) only	641	27.9%	0.0%	0.0%	0.0%	23.9%	17.0%	0.0%	31.2%

**Table 6:** The heat map shows frequencies of behaviours directed by the focus animal towards conspecifics and directed to the focus animal in the last month as reported by participating caretakers. Frequencies are depicted in the percentage of responses (*n*). The shade of orange darkens with increasing percentages.

Behaviours Directed Towards Conspecifics or Directed Towards Focus Animal	*n*	Never	1×/ Month	Several Times/Month	1×/ Week	Several Times/Week	1×/ Day	Several Times/ Day
Bites conspecifics	898	87.6%	4.9%	3.5%	1.2%	1.6%	0.8%	0.4%
Bitten by conspecifics	896	89.7%	5.4%	2.5%	0.6%	1.2%	0.2%	0.4%
Chases conspecifics	897	67.0%	14.0%	10.0%	2.9%	3.5%	1.2%	1.3%
Chased by conspecifics	892	70.3%	15.6%	7.7%	1.7%	1.9%	1.2%	1.6%
Blocks conspecifics from food	895	60.9%	8.8%	10.1%	2.5%	8.4%	4.2%	5.1%
Blocked from food by conspecifics	897	67.4%	8.7%	9.8%	2.1%	6.8%	2.3%	2.8%
Sprays urine at conspecifics	894	81.7%	8.6%	4.9%	1.1%	2.6%	0.7%	0.4%
Sprayed with urine by conspecifics	893	80.6%	8.5%	5.9%	1.2%	2.0%	0.8%	0.9%
Plucks out fur from conspecifics	895	96.9%	1.7%	0.9%	0.1%	0.4%	0.0%	0.0%
Fur plucked out by conspecifics	896	97.3%	1.7%	0.6%	0.0%	0.4%	0.0%	0.0%
Food stolen by conspecifics	892	32.3%	10.3%	14.8%	4.9%	17.0%	8.2%	12.4%
Steals food from conspecifics	894	29.0%	10.3%	15.9%	6.3%	17.0%	7.8%	13.8%
Mounts on conspecifics	891	63.0%	14.8%	12.1%	2.0%	5.7%	0.6%	1.8%
Mounted by conspecifics	887	66.0%	17.7%	9.8%	2.4%	2.7%	0.7%	0.8%
Plays with conspecifics	879	24.7%	4.9%	13.5%	2.6%	21.4%	5.7%	27.2%
Avoids contact with conspecifics	889	90.7%	3.6%	2.5%	0.3%	1.7%	0.3%	0.9%
Avoided by conspecifics	888	92.6%	2.7%	2.1%	0.6%	1.4%	0.3%	0.3%
Fighting with conspecifics	891	90.9%	5.3%	1.9%	0.7%	0.7%	0.6%	0.0%
Rests together with conspecific(s) (e.g., contact lying, sitting)	894	9.4%	3.7%	7.8%	1.7%	22.5%	6.8%	48.1%
Rests alone	888	8.1%	2.3%	8.1%	1.4%	19.3%	7.9%	53.0%
Naso-nasal contact	883	13.5%	4.3%	12.6%	3.5%	23.9%	8.6%	33.6%
Ano-genital sniffing (control)	881	14.1%	7.3%	16.7%	5.2%	25.2%	8.4%	23.2%
Sleeps together with conspecifics in same house/tube	892	18.0%	5.6%	11.1%	2.6%	19.2%	7.5%	36.0%
Eats simultaneously and peacefully next to conspecifics	895	0.6%	0.3%	1.8%	0.2%	6.8%	2.7%	87.6%

**Table 7:** Final stepwise linear regression model for the reported frequency of affiliative behaviours, and final stepwise logistic regression models for the occurrence of agonistic behaviours and competition for food. Behaviour subscales were calculated following a principal component analysis. For affiliative behaviours, subscale scores were obtained by calculating the mean of the items in the subscale. For agonistic behaviours and competition for food, subscale scores were dichotomized for the logistic regression models. Trends (*p* > 0.05 ≤ 0.1) are depicted in italics.

Dependent Variable	Predictor Variables and Model Summary	Estimate ^a^	SE ^b^	Beta ^c^	t	*p* ^d^
Frequency of affiliative behaviours	Number of permanently accessible huts/caves/other	0.11	0.06	0.07	1.82	*0.070*
	Group size increase within the prior 8 weeks no (0) yes (1)	−0.38	0.12	−0.12	−3.12	0.002
	Food enrichment	0.22	0.05	0.18	4.55	<0.001
	Daily time spent engaging with focus animal	0.10	0.05	0.07	1.89	*0.059*
	Frequency of carrying and stroking	0.07	0.02	0.12	3.21	0.001
	Frequency of talking hand-feeding	0.07	0.04	0.07	1.84	*0.067*
	Age of focus animal	−0.04	0.02	−0.06	−1.74	*0.081*
	Female (0) vs. male (1)	0.23	0.09	0.09	2.52	0.012
	Model: adj. R^2^ = 0.104, F = 10.78, *p* < 0.001, *n* = 674					
**Dependent Variables**	**Predictor Variables and Model Summary**	**B ^e^**	**SE**	**OR ^f^**	**Wald**	***p* ^d^**
				**(95% CI) ^g^**		
Occurrence of agonistic behaviours	Food enrichment	−0.15	0.08	0.86 (0.73–1.01)	3.55	*0.060*
	Model: R^2^ = 0.007, Chi^2^ = 3.61, *p* = 0.058, *n* = 676					
Occurrence of competition for food	Frequency of carrying and stroking	−0.17	0.47	0.84 (0.77–0.93)	12.92	<0.001
	Frequency of talking hand-feeding	0.26	0.08	1.30 (1.11–1.52)	10.73	0.001
	Female (0) vs. male (1)	−0.45	0.21	0.64 (0.43–0.96)	4.62	0.032
	Model: R^2^ = 0.056, Chi^2^ = 23.05, *p* < 0.001, *n* = 676					

^a^ Estimate: estimated regression coefficient (linear regression). ^b^ SE: standard error of estimate. ^c^ Beta: standardised regression coefficient (linear regression). ^d^ significance value. ^e^ B: regression coefficient (logistic regression). ^f^ OR: odds ratio. ^g^ 95% CI: 95% confidence interval of the lower and upper bounds of the OR.

**Table 8:** Heat map showing the frequency of behaviours displayed by focus animals in the main living area and during roaming in the last month. Frequencies are depicted in the percentage of responses (*n*). The shade of orange darkens with increasing percentages.

Behaviour Displayed in the Main Living Area and During Roaming	*n*	Never	1×/Month	Several Times/Month	1×/Week	Several Times/Week	1×/Day	Several Times/Day
Fur nibbling	913	67.0%	5.4%	7.2%	3.3%	7.4%	3.5%	6.1%
Bar chewing	913	96.7%	0.9%	0.5%	0.4%	0.3%	0.4%	0.7%
Running up and down at a certain cage location/between two specific places	919	91.1%	1.4%	1.2%	0.2%	2.4%	0.8%	2.9%
Urine spraying	913	80.2%	6.1%	6.5%	1.8%	3.9%	0.3%	1.2%
Rubbing anal region over the floor/marking with perianal glands	913	68.7%	5.8%	8.3%	2.4%	9.1%	1.8%	3.9%
Teeth grinding	909	78.0%	6.6%	6.6%	1.9%	4.1%	1.0%	1.9%
Teeth chattering (threatening behaviour)	912	50.3%	16.2%	14.8%	4.1%	8.7%	3.0%	3.0%
Gnawing furniture during free roaming	838	87.5%	3.3%	4.3%	1.1%	2.1%	1.0%	0.7%
Hiding during free roaming	802	48.9%	5.0%	8.4%	2.6%	10.5%	4.5%	20.2%
Trying to return to cage/enclosure during free roaming	784	73.6%	2.4%	4.6%	1.9%	3.7%	1.8%	12.0%
Popcorning (“jumping attacks”)	895	16.6%	8.4%	18.0%	4.9%	25.3%	6.9%	19.9%
Using toys/enrichment (e.g., intelligence toys, tunnels)	883	24.6%	4.1%	9.4%	2.7%	17.8%	6.0%	35.4%

**Table 9:** Differences in reported behaviours between the housing types (self-built enclosure, larger fenced floor area, free flat housing, and outdoor enclosure), according to post hoc testing (Mann–Whitney U tests) in case of overall significant difference in Kruskal–Wallis tests. Significant differences are marked in bold.

	Housing Types				
Housing Types	Self-Built Enclosure	Larger Fenced Floor Area	Guinea Pig Room	Free Housing	Outdoor Enclosure
**Frequency of Marking Behaviours and Teeth Noises**					
**Cage**	Z = −0.27 *p* = 0.791	Z = −0.73 *p* = 0.466	Z = −1.63 *p* = 0.103	Z = −1.10 *p* = 0.272	**Z = −3.20** ***p* = 0.001**
**Self-built enclosure**		Z = −0.64 *p* = 0.523	**Z = −1.97** ***p* = 0.048**	Z = −1.31 *p* = 0.189	**Z = −4.53** ***p* = <0.001**
**Larger fenced floor area**			**Z = −2.16** ***p* = 0.031**	Z = −1.51 *p* = 0.132	**Z = −4.43** ***p* = <0.001**
**Guinea pig room**				Z = −0.11 *p* = 0.909	Z = −0.42 *p* = 0.672
**Free housing**					Z = −0.42 *p* = 0.674
**Frequency of Going Back into the Enclosure and Hiding During Roaming**					
**Cage**	**Z = −2.16** ***p* = 0.031**	**Z = −2.06** ***p* = 0.039**	Z = −0.97 *p* = 0.333	Z = −1.16 *p* = 0.248	Z = −0.85 *p* = 0.398
**Self**-**built enclosure**		Z = −0.22 *p* = 0.825	Z = −0.15 *p* = 0.878	**Z = −2–12** ***p* = 0.034**	**Z = −3.36** ***p* = 0.001**
**Larger fenced floor area**			Z = −0.29 *p* = 0.775	**Z = −2.15** ***p* = 0.031**	**Z = −3.06** ***p* = 0.002**
**Guinea pig room**				Z = −1.58 *p* = 0.114	Z = −1.48 *p* = 0.138
**Free housing**					Z = −0.74 *p* = 0.458
**Frequency of Running Up and Down and Bar Chewing**					
**Cage**	**Z = 4.79** ***p* < 0.001**	**Z = −3.71** ***p* < 0.001**	**Z = −3.54** ***p* = <0.001**	**Z = −2.07** ***p* = 0.038**	**Z = −5.42** ***p* = <0.001**
**Self-built enclosure**		Z = −0.64 *p* = 0.525	**Z = −2.02** ***p* = 0.043**	Z = −0.70 *p* = 0.486	**Z = −2.09** ***p* = 0.036**
**Larger fenced floor area**			**Z = −2.23** ***p* = 0.026**	Z = −0.88 *p* = 0.378	**Z = −2.46** ***p* = 0.014**
**Guinea pig room**				Z = −1.36 *p* = 0.174	Z = −1.34 *p* = 0.180
**Free housing**					Z = −0.08 *p* = 0.939

**Table 10:** Final stepwise linear regression models for the reported frequency of going back into enclosure and hiding during roaming and the frequency of locomotor play and use of enrichment, and final stepwise logistic regression models for the occurrence of marking behaviours and teeth noises and running up and down and bar chewing. Behaviour subscales were calculated following a principal component analysis. For going back into the enclosure and hiding during roaming and the frequency of locomotor play and use of enrichment, subscale scores were obtained by calculating the mean of the items in the subscale. For the dependent variables of the logistics regression models, subscale scores were dichotomized (occurrence no/yes). Trends (*p* > 0.05 ≤ 0.1) are depicted in italics.

Dependent Variables	Predictor Variables and Model Summary	Estimate ^a^	SE ^b^	Beta ^c^	t	*p* ^d^
Frequency of going back into enclosure and hiding during roaming	Number of animals in the same enclosure as the focus animal	−0.03	0.02	−0.09	−2.22	0.027
	Roaming frequency outside the enclosure	0.15	0.02	0.38	9.72	<0.001
	Frequency of health checks	−0.25	0.09	−0.11	−2.83	0.005
	Model: adj. R^2^ = 0.151, F = 34.27, *p* < 0.001, *n* = 560					
Frequency of locomotor play and use of enrichment	Number of animals in the same enclosure as the focus animal	−0.04	0.01	−0.10	−2.65	0.008
	Food enrichment	0.33	0.07	0.18	4.72	<0.001
	Daily time spent engaging with focus animal	0.13	0.08	0.07	1.80	*0.073*
	Frequency of talking hand-feeding	0.21	0.06	0.14	3.60	<0.001
	Frequency of training	0.26	0.08	0.12	3.08	0.002
	Frequency of health checks	0.31	0.08	0.15	3.87	<0.001
	Age of focus animal	−0.21	0.03	−0.22	−6.02	<0.001
	Model: adj. R^2^ = 0.206, F = 23.49, *p* < 0.001, *n* = 609					
**Dependent Variables**	**Predictor Variables and Model Summary**	**B ^e^**	**SE**	**OR ^f^**	**Wald**	***p* ^d^**
				**(95% CI) ^g^**		
Observation of marking behaviour and teeth noises	Daily time spent engaging with focus animal	0.22	0.12	1.25 (0.98–1.60)	3.31	*0.069*
	Frequency of training	0.36	0.16	1.43 (1.05–1.95)	5.06	0.025
	Frequency of talking hand-feeding	0.19	0.07	1.21 (1.05–1.39)	7.11	0.008
	Model: R^2^ = 0.057, Chi^2^ = 25.88, *p* < 0.001, *n* = 627					
Observation of running up and down and bar chewing	Individual housing yes (0) no (1)	−0.70	0.37	0.50 (0.24–1.04)	3.48	*0.062*
	Frequency of training	0.30	0.12	1.35 (1.07–1.72)	6.17	0.013
	Frequency of carrying and stroking	0.19	0.05	1.20 (1.08–1.34)	11.53	0.001
	Model: R^2^ = 0.069, Chi^2^ = 22.82, *p* < 0.001, *n* = 626					

^a^ Estimate: estimated regression coefficient (linear regression). ^b^ SE: standard error of the estimate. ^c^ Beta: standardised regression coefficient (linear regression). ^d^ significance value. ^e^ B: regression coefficient (logistic regression). ^f^ OR: odds ratio. ^g^ 95% CI: 95% confidence interval of the lower and upper bounds of the OR.

## Data Availability

Restrictions apply to the datasets. The datasets presented in this article are not readily available because they must not be shared with third parties and because of ongoing data analysis for follow-up manuscripts. Requests to access the datasets should be directed to [I. Windschnurer, ines.windschnurer@vetmeduni.ac.at].

## Supplementary Materials

The following supporting information can be downloaded at: https://www.mdpi.com/article/10.3390/ani15081157/s1, Table S1: Presence of dogs and cats in the same room as the focus animal and frequencies thereof; Table S2: Principal component analysis of the different enrichment items; Table S3: Time spent on average per day engaging with the focus animal; Table S4: Principal component analysis of human–animal interactions; Table S5: Principal component analysis of (health) care measures; Table S6: Principal component analysis of behaviours displayed in a social context; Table S7: Principal component analysis of behaviours in the main living area; Table S8: Frequencies of behaviours across housing types observed in the main living area and during roaming.
