# Supplementary material for: Guinea Pig (Cavia porcellus) Welfare: Associations Between Husbandry Practices, Human–Animal Interactions, and Animal Behaviour
Source: Animals (Basel). 2025 Apr 17;15(8):1157. doi: 10.3390/ani15081157 (PMC12024334; doi:10.3390/ani15081157)
Supplement: Supplementary file 1 [file animals-15-01157-s001.zip › animals-3530837-supplementary.pdf]

Supplementary Materials

Guinea pig (*Cavia porcellus*) welfare: associations between husbandry practices, human-animal interactions, and animal behaviour

Tanja Elsbacher [a], Andrea Sommese [a], Susanne Waiblinger[a], Frank Künzel[b], Christine Arhant[a,c], Ines Windschnurer[a]*

[a] Centre for Animal Nutrition and Welfare, Clinical Department for Farm Animals and Food System Science, University of Veterinary Medicine Vienna, Veterinärplatz 1, 1210 Vienna, Austria;

[b] Clinical Unit of Internal Medicine Small Animals, Special ambulance for small mammals, University of Veterinary Medicine Vienna, Veterinärplatz 1, 1210 Vienna, Austria;

[c] Tierärztliche Praxis für Verhaltensmedizin Tierfairhalten, Gwörth 2, 3073 Kasten, Austria

e-mail: elsbacher_tanja@hotmail.com, andrea.sommese@vetmeduni.ac.at, ines.windschnurer@vetmeduni.ac.at, susanne.waiblinger@vetmeduni.ac.at, frank.kuenzel@vetmeduni.ac.at, ch.arhant@gmail.com

*Correspondence: ines.windschnurer@vetmeduni.ac.at; Tel: 0043 1 25077 4915

Table S1

Presence of dogs and cats in the same room as the focus animal and frequencies thereof in percentage of responses.

**Frequency of presence of dogs (n=983 responses)**	**n**	**%**
Never	655	66.6
Less often than 1x/week	73	7.4
1x/week	8	0.8
Several times per week	23	2.3
1x/day	11	1.1
Several times/day	115	11.7
Constantly	98	10.0
**Frequency of presence of cats (n=926 responses)**	**n**	**%**
Never	739	79.8
Less often than 1x/week	24	2.6
1x/week	6	0.6
Several times per week	12	1.3
1x/day	8	0.9
Several times/day	80	8.6
Constantly	57	6.2

Table S2

Principal component analysis of the different enrichment items offered based on n = 871 responses. The Kaiser-Meyer-Olkin (KMO) measure of sampling adequacy was 0.576, and Bartlett's test for sphericity was significant (χ^2 = 248.07, $p < 0.001$). Loadings below 0.3 are not depicted in the rotated component matrix. Carton boxes and tunnels were not included in the final PCA because their component loadings did not reach 0.4. Food balls and intelligence toys were excluded because of double loadings that were too close (did not exceed 0.6 for one component while being below 0.4 for the other.

**Enrichment Items Offered**	**Components and Component Loadings**	
	**Food Enrichment**	**Cat and Dog Toys**
Hay ball	0.71	
Gnawing stick from wood	0.67	
Feeding tree	0.52	
Fresh twigs	0.51	
Cat toys		0.77
Dog toys		0.76
% of variance explained by component	24.77	22.91
Cronbach Alpha	0.44	0.46

Extraction method: principal component analysis.
Rotation method: Varimax rotation.

Table S3

Time spent on average per day engaging with the focus animal (hand-feeding, stroking, playing, training, observing, etc.) as reported by 1060 caretakers.

**Daily time spent engaging with focus animal (hours)**	**n**	**%**
0	28	2.6
0.5	516	48.7
1	242	22.8
1.5	92	8.7
2	86	8.1
2.5	26	2.5
3	26	2.5
3.5	8	0.8
4	16	1.5
4.5	3	0.3
5	4	0.4
5.5	4	0.4
6	4	0.4
6.5	0	0
7.0	2	0.2
7.5	0	0
8.0	2	0.2
8.5	0	0
9	0	0
9.5	0	0
10.0	1	0.1

Table S4

Principal component analysis of human-animal interactions as reported by caretakers based on n = 903 responses. The Kaiser-Meyer-Olkin criterion of sampling adequacy was 0.588 and Bartlett's test for sphericity was significant ($\chi^2 = 952.11$, $p < 0.001$). Loadings below 0.3 are not depicted in the rotated component matrix.

**Human–animal interactions**	**Components and Component loadings**		
	**Frequency of Training**	**Frequency of Carrying and Stroking**	**Frequency of Talking and Hand-feeding**
Target training	**0.81**		
Clicker training	**0.80**		
Trick training	**0.54**		
Agility	**0.495**		
Carrying around		**0.89**	
Stroking		**0.82**	
Talking			**0.81**
Hand-feeding			**0.76**
% of variance explained by component	23.29	18.66	17.21
Cronbach Alpha	0.56	0.65	0.46

Extraction method: principal component analysis.
Rotation method: Varimax rotation.

Table S5

Principal component analysis of (health) care measures as reported by caretakers based on n = 912 responses. Data were suitable for PCA, as confirmed by a Kaiser-Meyer-Olkin criterion of sampling adequacy of 0.721 and Bartlett's test for sphericity being significant (Chi2 = 2824.03, $p < 0.001$). Loadings below 0.3 are not depicted in the rotated component matrix. Nail clipping was not included in the final PCA because the component loading did not reach the threshold of 0.4.

**(Health) Care Measures**	**Components and Component loadings**	
	**Frequency of Health Checks**	**Frequency of Cleaning and Fur Care**
Anterior teeth check	0.88	
Ear check	0.87	
Anal region check	0.81	
Cleaning nasal region		0.93
Cleaning eye region		0.92
Fur grooming		0.57
% of variance explained by component	38.32	35.76
Cronbach Alpha	0.85	0.78

Extraction method: principal component analysis.
Rotation method: Varimax rotation.

Table S6

Principal component analysis of behaviours displayed in a social context based on n = 833 responses. The Kaiser-Meyer-Olkin criterion of sampling adequacy was 0.631 and Bartlett's test for sphericity was significant (Chi2 = 3627.36, $p < 0.001$). Loadings below 0.3 are not depicted in the rotated component matrix.

**Behaviours directed towards conspecifics and directed to focus animal by conspecifics**	**Components and Component loadings**		
	**Frequency of Agonistic Behaviours**	**Frequency of Affiliative Behaviours**	**Frequency of Competition for Food**
hunts conspecifics	0.72		
bitten by conspecifics	0.71		
bites conspecifics	0.70		
hunted by conspecifics	0.69		
fighting with conspecifics	0.66		
resting with conspecifics		0.72	
naso-nasal contact		0.69	
sleeping with conspecific(s) in same house/tube		0.63	
plays with conspecifics		0.625	
anogenital control		0.58	
eat together		0.48	
steals food from conspecifics			0.82
food stolen by conspecifics			0.80
blocked from food by conspecifics			0.71
blocks conspecifics from food			0.67
% of variance explained by component	16.25	15.11	15.00
Cronbach Alpha	0.73	0.68	0.76

Extraction method: principal component analysis.
Rotation method: Varimax rotation.

Table S7

Principal component analysis of behaviours in the main living area based on n = 728 responses. The Kaiser-Meyer-Olkin criterion of sampling adequacy was 0.638 and Bartlett's test for sphericity was significant (Chi2 = 718.32, $p < 0.001$). Loadings below 0.3 are not depicted in the rotated component matrix. Fur nibbling and gnawing at furniture during roaming were not included in the final PCA because their component loadings did not reach the threshold of 0.4.

**Behaviours in the main living area and during roaming**	**Components and Component loadings**			
	**Frequency of Marking and Teeth Noises**	**Frequency of Going Back into Enclosure and Hiding During Roaming**	**Frequency of Running up and down and Bar Chewing**	**Frequency of Locomotor Play and Use of Enrichment**
Rubbing anal region over the floor/marking with perianal glands	0.75			
Urine spraying	0.70			
Teeth grinding	0.63			
Teeth chattering (threatening behaviour)	0.63			
Going back into the enclosure during free-roaming		0.86		
Hiding during free-roaming		0.84		
Bar chewing			0.80	
Running up and down at a certain cage location/between two specific places			0.74	
Popcorning ('jumping attacks')				0.79
Using toys/enrichment (e.g. intelligence toys, tunnels)				0.78
% of variance explained by component	18.89	14.81	13.02	12.80
Cronbach Alpha	0.61	0.62	0.31	0.42

Extraction method: principal component analysis.
Rotation method: Varimax rotation.

Table S8

Frequencies of behaviours across housing types observed in the main living area and during roaming within the last month before the survey. Behaviours were summarized through PCA calculating mean score across summarized items. The score could range from 1 (never) to 7 (several times per day).

**Frequency score for…**	**Housing types**																							
	**Cage**								**Self-built enclosure**								**Larger fenced floor area**							
	**n**	**mean**	**SD**	**Min**	**25**	**med**	**75**	**max**	**n**	**mean**	**SD**	**min**	**25**	**med**	**75**	**max**	**n**	**mean**	**SD**	**min**	**25**	**med**	**75**	**max**
**Marking behaviours & teeth noises**	109	1.90	1.11	1	1	1.5	2.3	7.0	387	1.90	1.03	1	1	1.5	2.5	6.5	199	2.00	1.15	1	1	1.50	2.5	6.0
**Going back into enclosure & hiding during free-roaming**	104	2.83	1.90	1	1	2.0	4.0	7.0	332	2.45	1.85	1	1	1.5	3.5	7.0	164	2.40	1.83	1	1	1.25	3.5	7.0
**Running up and down & bar chewing**	109	1.68	1.28	1	1	1.0	1.5	7.0	387	1.22	0.79	1	1	1.0	1.0	7.0	198	1.18	0.59	1	1	1.00	1.0	4.0
**Using toys & enrichment**	108	4.36	1.86	1	3	4.5	6.0	7.0	381	4.27	1.82	1	3	4.0	6.0	7.0	195	4.47	1.76	1	3	4.50	6.0	7.0
**Frequency score for…**	**Guinea pig room**								**Free flat housing**								**Outdoor housing**							
	**n**	**mean**	**SD**	**min**	**25**	**med**		**max**	**n**	**mean**	**SD**	**min**	**25**	**med**	**75**	**max**	**n**	**mean**	**SD**	**min**	**25**	**med**	**75**	**max**
**Marking behaviours & teeth noises**	37	1.57	0.77	1	1	1.3	2.0	4.5	20	1.63	0.84	1	1	1.3	2.0	4.0	170	1.49	0.67	1	1.0	1.3	1.75	4.00
**Going back into enclosure & hiding during free-roaming**	35	2.60	2.13	1	1	1.5	4.0	7.0	20	3.58	2.52	1	1	3.0	7.0	7.0	159	3.18	2.23	1	1.0	3.0	4.00	7.00
**Running up and down & bar chewing**	37	1.00	0.00	1	1	1.0	1.0	1.0	20	1.15	0.67	1	1	1.0	1.0	4.0	170	1.11	0.59	1	1.0	1.0	1.00	6.50
**Using toys & enrichment**	36	3.96	1.75	1	3	4.0	5.0	7.0	20	4.93	1.21	3	4	5.0	6.0	7.0	165	4.07	1.89	1	2.5	4.0	6.00	7.00
